# SDG26 integrates ABA‐dependent and ABA‐independent pathways to regulate cold stress responses in Arabidopsis via chromatin modification

**DOI:** 10.1111/tpj.71180

**Published:** 2026-10-23

**Authors:** Xue Zhang, Julie Lapierre, Julie Zumsteg, Mathieu Erhardt, Wen‐Hui Shen, Alexandre Berr

**Affiliations:** ^1^ Institut de Biologie Moléculaire des Plantes du CNRS Université de Strasbourg 12 rue du Général Zimmer Strasbourg Cedex 67084 France; ^2^ Université de Strasbourg, CNRS, IPHC UMR7178 Strasbourg F‐67000 France; ^3^ Present address: Division of Genome Biology, Department of Medical Biochemistry and Biophysics Karolinska Institutet SE‐17177 Stockholm Sweden; ^4^ Present address: Science for Life Laboratory Sweden, Tomtebodavägen 23A 17165 Solna Sweden

**Keywords:** *Arabidopsis thaliana*, histone methylation, SDG26, abscisic acid, abiotic stress, cold stress

## Abstract

Plants constantly face adverse environmental conditions, with temperature drops posing major threats to growth and productivity. To adapt, plants have evolved complex mechanisms of transcriptional reprogramming. While various cold‐responsive pathways have been described, the contribution of chromatin‐level regulation, particularly histone modifications, remains poorly understood. Here, we identify the histone methyltransferase SET DOMAIN GROUP 26 (SDG26) as a positive regulator of cold stress responses in *Arabidopsis thaliana*. We show that *SDG26* is transcriptionally induced and posttranscriptionally stabilized by cold, and that its loss of function compromises several abscisic acid (ABA)‐related phenotypes. At the molecular level, SDG26 promotes the expression of cold‐responsive genes, including members of the CBF‐COR regulon, through direct chromatin binding and deposition of histone H3 lysine 36 trimethylation (H3K36me3). Additionally, SDG26 modulates ABA biosynthesis, catabolism, and transport, thereby promoting ABA accumulation, stomatal closure, and drought tolerance. Collectively, our results reveal that SDG26 integrates ABA‐dependent and ABA‐independent pathways to fine‐tune Arabidopsis responses to abiotic stresses. We further establish SDG26 as a chromatin modifier contributing to stress‐responsive H3K36me3 enrichment at specific loci. Together, our findings position SDG26 as a chromatin‐based hub that coordinates cold acclimation and water conservation to enhance plant resilience.

## Significance Statement

Plants must rapidly reprogram their gene expression to survive cold, yet how chromatin marks contribute to this response remains poorly understood. We show that the histone methyltransferase SDG26 integrates ABA‐dependent and ABA‐independent signaling to coordinate cold acclimation, stomatal closure, drought tolerance, and seed dormancy in Arabidopsis, establishing it as a chromatin‐based hub linking hormonal and environmental stress responses.

## INTRODUCTION

Plants, as sessile organisms, must continuously adapt to fluctuating environmental conditions and withstand diverse stressors throughout their life cycle. Exposure to these stressors elicits extensive physiological, biochemical, and molecular changes that can severely constrain plant growth and pose a threat to agricultural productivity (Zhang et al., 2022). Epigenetic mechanisms, which provide more rapid and reversible responses compared to genetic mutations, have been notably implicated in environmental stress responses, adaptation, and memory in plants (Gaude et al., 2024). Among them, histone lysine methylation has emerged as a central regulatory axis that modulates chromatin accessibility. Histone lysine methylation is catalyzed by histone lysine methyltransferases (HKMTs) and does not directly alter chromatin conformations but instead serves as a recruitment platform for specific “readers” that shape transcriptional outcomes in a context‐dependent manner (Hu & Du, 2022). Depending on the lysine residue targeted and the number of methyl groups added (mono‐, di‐, or tri‐methyl), this versatile posttranslational modification can exert dual effects on gene transcription, either activating or repressing it. In *Arabidopsis thaliana*, approximately 40 putative HKMTs have been identified and grouped into five classes based on sequence homology (Springer et al., 2003). The SET DOMAIN GROUP (SDG) family includes ABSENT, SMALL OR HOMEOTIC DISCS1 (ASH1) and TRITHORAX (TRX)‐related enzymes, which typically associate with transcriptional activation via H3K4 and/or H3K36 methylation. Within this family, SDG26/ASHH1 has been primarily studied for its role in flowering‐time control. Unlike mutants of its homologs SDG8/ASHH2 (Zhao et al., 2005) or *SDG7/ASHH3* (Lee et al., 2015), or other TRX subfamily members such as *ATX1/SDG27* (Pien et al., 2008), *SDG2/ATRX3* (Berr, McCallum, Alioua, et al., 2010; Berr, McCallum, Ménard, et al., 2010; Guo et al., 2010), and *SDG25/ATXR7* (Berr et al., 2009; Tamada et al., 2009), the *sdg26* mutant exhibits a unique late‐flowering phenotype (Xu et al., 2008). Notably, even *CURLY LEAF* (*CLF*), a member of the repressive E(z) family, produces an early‐flowering phenotype when mutated (Jiang et al., 2008).

SDG8 delays flowering by activating the transcription of the floral repressor *FLOWERING LOCUS C* (*FLC*) through H3K36 di‐ and tri‐methylation (Xu et al., 2008). In contrast, SDG26 participates in a multi‐subunit complex involving autonomous pathway components such as the H3K4 demethylase FLOWERING LOCUS D (FLD), the transcriptional repressor LUMINIDEPENDENS (LD), ELF4‐like proteins (EFL2 and EFL4), and the yeast COMPASS complex subunit SWD2 homolog APRF1/S2La (Fang et al., 2020; Qi et al., 2022). Within this complex, SDG26 links RNA 3′‐end processing to chromatin regulation, repressing *FLC* transcription (Xu et al., 2021). Beyond this repressive function, SDG26 also promotes transcriptional activation of the floral integrator *SUPPRESSOR OF OVEREXPRESSION OF CONSTANS1* (*SOC1*) through H3K4 and H3K36 trimethylation (Berr et al., 2015) (Liu, Berr, et al., 2016). These dual activities suggest that SDG26 is a versatile chromatin modifier that can act either as a repressor or activator depending on genomic context and/or interacting partners.

Although most studies of SDG26 have focused on floral transition, emerging evidence indicates that its functions extend further. SDG26 has been implicated in the regulation of pre‐messenger RNA splicing (Pajoro et al., 2017), trichome growth and development (Zeng et al., 2023), and the DNA damage response through mechanisms that remain to be defined (Campi et al., 2012) (Roitinger et al., 2015). Notably, previous transcriptomic analysis of *sdg26* mutants revealed that nearly one third of differentially expressed genes are linked to stress responses (Liu, Berr, et al., 2016) (Berr et al., 2015). *In silico* analysis suggests that SDG26 expression is stress‐responsive to cold, drought, and salt (Naika et al., 2013). Moreover, its promoter contains cis‐regulatory elements such as putative W‐box motifs, which are binding sites for WRKY transcription factors that mediate numerous biotic and abiotic stress responses (Phukan et al., 2016). Consistent with this, publicly available transcriptomic data from the Arabidopsis eFP Browser (Winter et al., 2007) show that *SDG26* transcript levels are induced in response to abiotic stresses (e.g., cold, drought and salt). Together, these observations raise the possibility that SDG26 functions beyond development, contributing to stress adaptation.

In this study, we demonstrate that SDG26 is a critical regulator of Arabidopsis responses to cold stress. We show that *SDG26* transcription is induced by cold, salt, and drought stress, and that its protein abundance is stabilized under cold conditions. At the molecular level, we reveal that *SDG26* modulates a subset of cold‐responsive genes through both the CBF‐COR pathway and abscisic acid (ABA) homeostasis. Loss of *SDG26* function impairs ABA accumulation, delays cold‐induced stomatal closure, and reduces seed dormancy, while paradoxically enhancing freezing tolerance but compromising drought resistance. Collectively, our findings establish SDG26 as a chromatin‐based integrator of ABA‐dependent and ABA‐independent pathways, highlighting its role in fine‐tuning stress adaptation and in coordinating responses to multiple abiotic challenges.

## RESULTS

### *SDG26* is induced by different abiotic stresses but not by stress‐related phytohormones

RT‐qPCR analysis revealed stable SDG26 transcript levels across organs and the diurnal cycle (Figure 1A,B). GUS staining and *in situ* hybridization confirmed SDG26 expression across organs and developmental stages, with particularly strong activity in vascular tissues, young and actively dividing tissues, and reproductive structures (Figures S1, S2). In addition, grafting experiments on *sdg26‐1* lines expressing a C‐terminal GFP‐tagged SDG26 under the 35S promoter (*sdg26‐1* p35S::SDG26‐GFP; Figure S3) demonstrated that SDG26 does not exhibit long‐distance mobility between scion and rootstock (Figure S4). Given this broad expression pattern, we next investigated whether SDG26 responds to abiotic stress. The presence of a W‐box motif in the SDG26 promoter, along with previous transcriptomic data suggesting stress responsiveness, prompted us to test its induction under various stress conditions. RT‐qPCR analysis showed that cold, drought, and salt treatments each triggered a modest but significant transient increase in *SDG26* transcript abundance (Figure 1C–E). In contrast, no significant induction occurred with stress‐related signaling molecules such as ABA, ACC, kinetin, salicylic acid, or methyl jasmonate (Figure S5). To confirm these results, we monitored GUS activity in the pSDG26‐GUS line described above. Cold treatment enhanced GUS staining compared with control conditions (Figure 1F), confirming our RT‐qPCR results. We then tested whether transcript accumulation translated into protein‐level changes. To this end, we generated *sdg26‐1* transgenic plants expressing SDG26 fused to a 10x c‐Myc tag under the control of its native promoter (p+gSDG26‐MYC; Figure S3). In these plants, immunoblot analysis revealed a gradual increase of SDG26 protein during cold exposure (Figure 1G). Importantly, subcellular localization remained unchanged, SDG26‐GFP localized exclusively to the nucleus under both control and cold stress conditions (Figure S6). Together, these results show that *SDG26* is induced by multiple abiotic stresses at both the transcription and protein levels, but not directly by stress‐related phytohormones. This suggests that SDG26 functions as an upstream chromatin regulator responding primarily to environmental cues rather than to hormonal signals.

**Figure 1:**
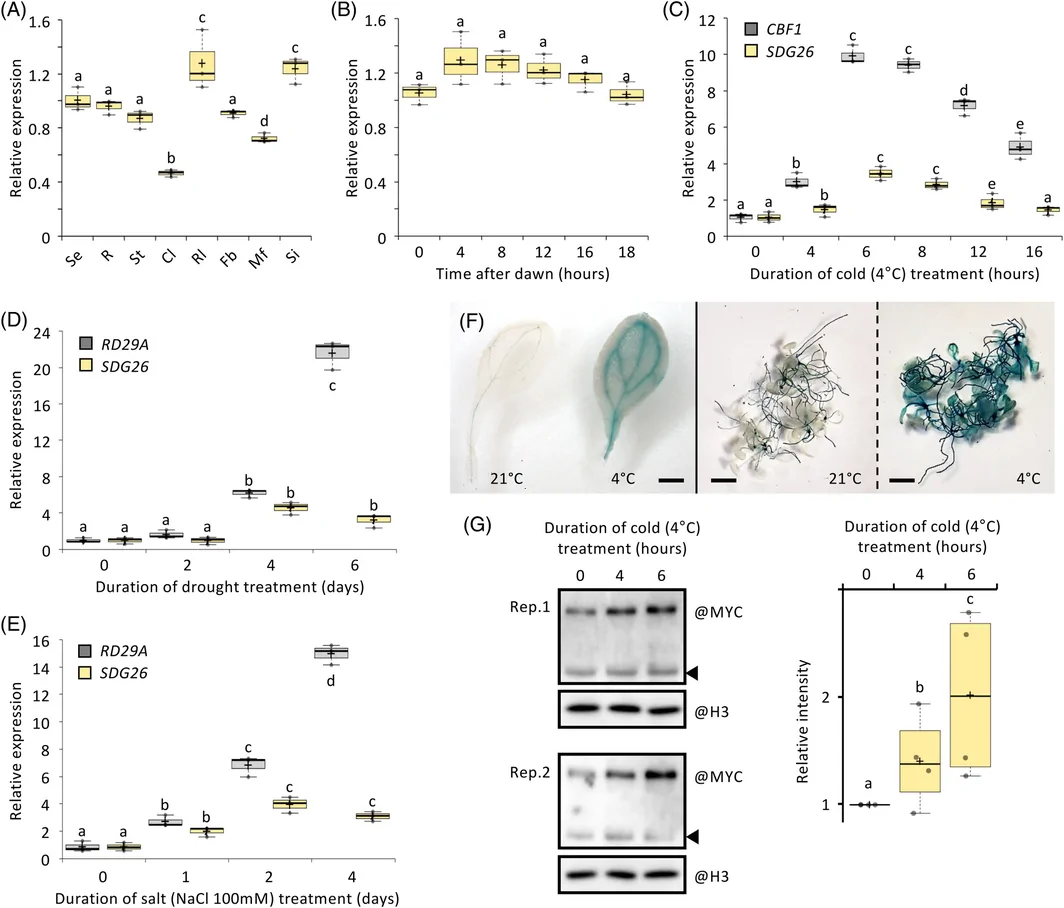
*SDG26* expression is induced by abiotic stresses at both transcript and protein levels. (A) Relative transcript levels of *SDG26* in different organs of wild‐type Col‐0 plants. Se: seedling; R: root; St: stem; Cl: cauline leaf; Rl: rosette leaf; Fb: flower bud; Mf: mature flower; Si: silique. (B) Transcript levels of *SDG26* in 2‐week‐old seedlings grown under long‐day (LD; 16 h light/8 h dark) conditions, sampled at indicated times after dawn. (C–E) Transcript levels of *SDG26* and a stress‐responsive reference gene (*CBF1* for cold, *RD29A* for drought and salt) in Col‐0 plantlets subjected to (C) cold stress (4°C), (D) drought stress, or (E) salt stress (NaCl 100 mM) for the indicated durations. Transcript levels are expressed relative to the transcript level in seedlings for (A) or at time 0 h after dawn for (B). (F) Representative GUS staining images of *pSDG26‐GUS* seedlings maintained at 21°C or transferred to 4°C for 4 h. Stressed (4°C) and non‐stressed (21°C) transgenic lines were similarly fixed in 80% acetone and stained at 37°C for 6 h. Bars = 0.5 cm (left) and 1 cm (right). (G) Left: immunoblot detection of SDG26‐Myc protein in the *sdg26‐1 p + gSDG26‐Myc* transgenic line before (0 h) and after 4 or 6 h of cold treatment (4°C), using an anti‐Myc antibody. Histone H3 and a nonspecific band (arrowhead) were used for normalization. Two biological replicates (Rep.1 and Rep.2) are shown. Right: densitometric quantification of SDG26‐Myc band intensity relative to time 0 (set as 1). In all panels, data are presented as box plots showing median, mean (+), and individual data points from at least three biological replicates. Letters indicate significant differences (Student's *t*‐test with Benjamini–Hochberg FDR correction, *P* < 0.05).

### SDG26 directly binds cold‐related genes and regulates their transcription

SDG26 was previously shown to directly regulate *SOC1* expression in flowering‐time control (Berr et al., 2015). Because *SOC1* also participates in a feedback loop linking flowering and cold response (Seo et al., 2009), we examined its regulation by SDG26 during cold stress. Under control conditions (21°C), *SOC1* expression remained relatively stable, while upon cold exposure (4°C), *SOC1* transcript levels gradually increased in both Col‐0 and *sdg26‐1* plants, as well as in our p+gSDG26‐Myc transgenic line (Figure 2A; Figure S7). Interestingly, these increases were diminished in *sdg26‐1* compared to Col‐0. To investigate how *SDG26* regulates *SOC1* expression, we performed ChIP‐qPCR to assess changes in H3K4me3 and H3K36me3 levels, two active histone marks, at the *SOC1* chromatin (Figure 2B). As previously reported (Berr et al., 2015; Liu, Berr, et al., 2016; Liu, Wei, et al., 2016), H3K36me3 and H3K4me3 levels were lower in *sdg26‐1* compared to Col‐0 plants grown at 21°C. Upon cold exposure, H3K36me3 levels remained unchanged in both Col‐0 and *sdg26‐1*, whereas H3K4me3 levels increased (Figure 2B). To determine whether SDG26 is recruited to SOC1 chromatin in response to cold, we performed ChIP assays using two complementary approaches: (*ii*) a MYC antibody on chromatin extracts from both non‐transformed Col‐0 plants and p+gSDG26‐Myc transgenic line, and (*i*) a monoclonal SDG26 antibody on Col‐0 and *sdg26‐1* chromatin extracts. In both cases, SDG26 occupancy at SOC1 chromatin remained unchanged following cold stress (Figure 2B). Thus, SDG26 contributes both to *SOC1* basal expression and to its cold‐induced upregulation, without changes in recruitment.

**Figure 2:**
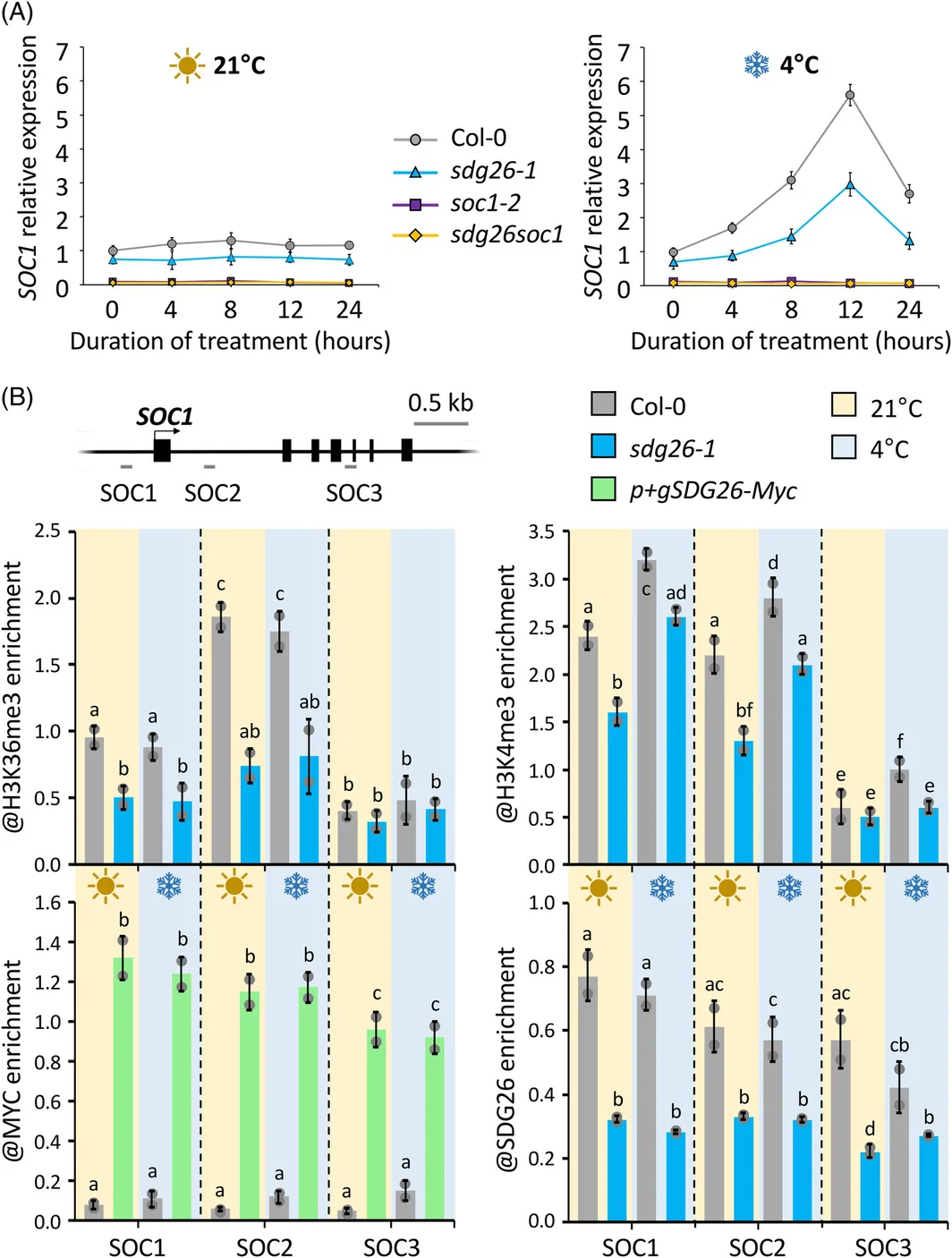
Transcript and chromatin analysis of *SOC1* during cold stress. (A) Dynamic expression of *SOC1* in 2‐week‐old Col‐0, *sdg26‐1*, *soc1‐2*, and *sdg26soc1* seedlings at 21°C (left) or subjected to cold stress at 4°C (right) for the indicated durations. Data are relative to Col‐0 at 0 h (set to 1) and are shown as mean ± SD of three biological replicates. (B) Top: schematic structure of the *SOC1* locus, with black boxes representing exons, black lines representing the promoter and introns, an arrow indicating the translational start site, and gray bars indicating the amplified regions (SOC1, SOC2, SOC3). Bottom: ChIP‐qPCR analysis of H3K36me3 (top left) and H3K4me3 (top right) in Col‐0 and *sdg26‐1*, MYC enrichment in *sdg26‐1 p + gSDG26‐Myc* and Col‐0 (used as a negative control) (bottom left), and SDG26 enrichment in Col‐0 and *sdg26‐1* (used as a negative control) using an anti‐SDG26 monoclonal antibody (bottom right), along different regions of *SOC1* chromatin. Plant material was collected after 8 h of treatment at 21°C (sun symbol) or 4°C (snowflake symbol). Data are presented as the ratio of each mark or factor over H3 for each amplified region. Mean values ± SD are presented based on results from two biological replicates. Letters indicate significant differences (Student's *t*‐test with Benjamini–Hochberg FDR correction, *P* < 0.05).

SOC1 is a known negative regulator of the cold‐responsive CBF genes *CBF1*, *CBF2*, and *CBF3*, and their downstream targets, the CBF‐target genes *COR15A*, *RD29A*, *KIN1*, and *KIN2* (Seo et al., 2009). Given that *SDG26* regulates *SOC1* transcription and is itself induced by cold, we next examined whether SDG26 also affects the expression of cold‐responsive genes. Consistent with previous reports (Seo et al., 2009), *CBF* genes and their downstream targets were upregulated in *soc1‐2* compared to Col‐0 under both control and cold conditions (Figure 3A; Figure S8). Surprisingly, despite the lower *SOC1* expression observed in *sdg26‐1* and *sdg26soc1* (Figure 2A), *CBF* and its target genes showed weaker induction by cold compared to Col‐0 (Figure 3A). To resolve this paradox, we tested whether SDG26 directly regulates CBF genes. ChIP‐qPCR revealed SDG26 enrichment at CBF1‐3 chromatin, increased under cold treatment (Figure 3B). In parallel, H3K36me3 levels at *CBF1* were reduced in *sdg26‐1* relative to Col‐0 under control conditions and showed impaired increase upon cold exposure (Figure 3B). These results suggest that SDG26 exerts a dual regulatory role by positively regulating *SOC1*, a repressor of CBFs, while simultaneously directly activating CBF loci through H3K36me3 deposition. This dual action suggests that SDG26 fine‐tunes the cold response by balancing repression via *SOC1* with direct activation of cold‐inducible genes.

**Figure 3:**
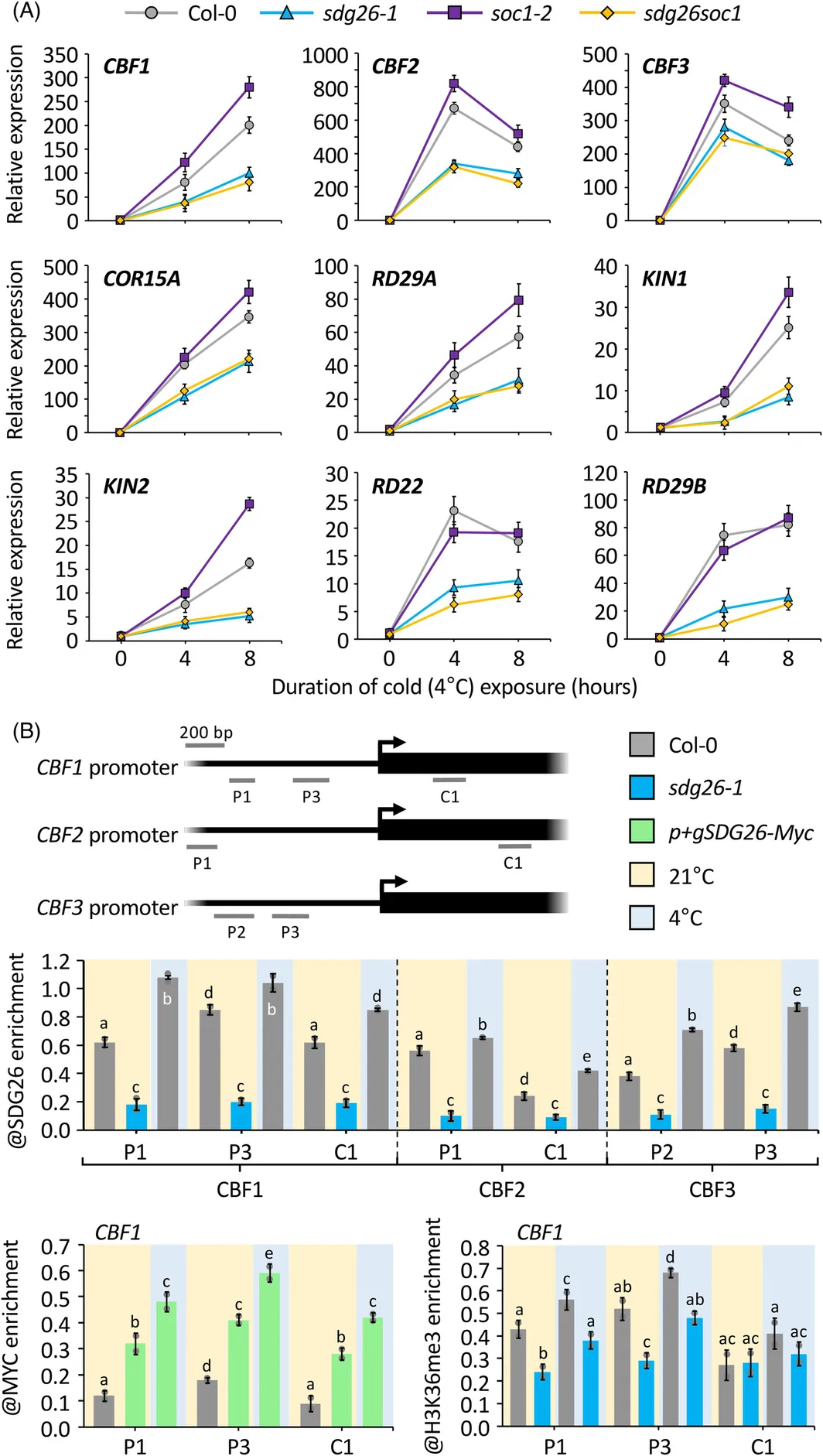
Transcript and chromatin analysis of cold‐inducible genes during cold stress. (A) Expression of cold‐inducible genes was determined in 2‐week‐old Col‐0, *sdg26‐1*, *soc1‐2* and *sdg26soc1* seedlings before (0 h) and during (4 and 8 h) a cold treatment at 4°C. Data are presented relative to Col‐0 at 0 h (set as 1) as mean ± SD of at least three biological replicates. (B) Top: schematic diagrams adapted from (Shi et al., 2012) showing the promoter structures of the *CBF1*, *CBF2*, and *CBF3*. The 1.0 kb upstream regions and coding regions are shown, with the translational start site as an arrow and amplified regions as gray lines. Bottom: analyses of the SDG26 enrichment at different regions of the *CBF1, CBF2, and CBF3* genes by ChIP using an anti‐SDG26 monoclonal antibody in Col‐0 treated with a cold stress (4°C) or not (21°C) and in *sdg26‐1* used as a negative control. SDG26 enrichment at the CBF1 locus was further confirmed using an anti‐MYC antibody in *sdg26‐1 p + gSDG26‐Myc* transgenic plants. The level of H3K36me3 along the *CBF1* chromatin was also measured by ChIP in plants treated with a cold stress (4°C) or not (21°C). Data are presented as the ratio of SDG26, SDG26‐MYC, or H3K36me3 over H3 for each amplified region. Mean values ± SD are presented based on results from two biological replicates. Letters indicate significant differences (Student's *t*‐test with Benjamini–Hochberg FDR correction, *P* < 0.05).

### SDG26 plays roles in ABA homeostasis

In addition to CBF‐dependent pathways, plants activate ABA‐dependent but CBF‐independent signaling during cold stress (Barnaby et al., 2020). Genes such as *RD29B* and *RD22* fall into this category (Zhang et al., 2004). Their expression was similarly induced in *soc1‐2* and Col‐0 plants but was markedly reduced in *sdg26‐1* and *sdg26soc1* mutants under cold conditions (Figure 3A; Figure S8). Given that ABA accumulates in response to cold stress (Pareek et al., 2017), this suggested a possible defect in ABA homeostasis. We therefore quantified ABA levels during cold exposure. The phytohormone jasmonate (JA), known to positively regulate cold stress responses (Hu et al., 2017), was also quantified. While JA levels remained unchanged across all tested genotypes, ABA accumulation was significantly lower in *sdg26‐1* and *sdg26soc1* than in Col‐0 and *soc1‐2* under both control and cold conditions (Figure 4A). This indicates that SDG26 regulates cold stress responses, at least in part, by promoting ABA accumulation, which could explain the lower expression of *RD29B* and *RD22* in *sdg26‐1* and *sdg26soc1* mutants upon cold exposure.

**Figure 4:**
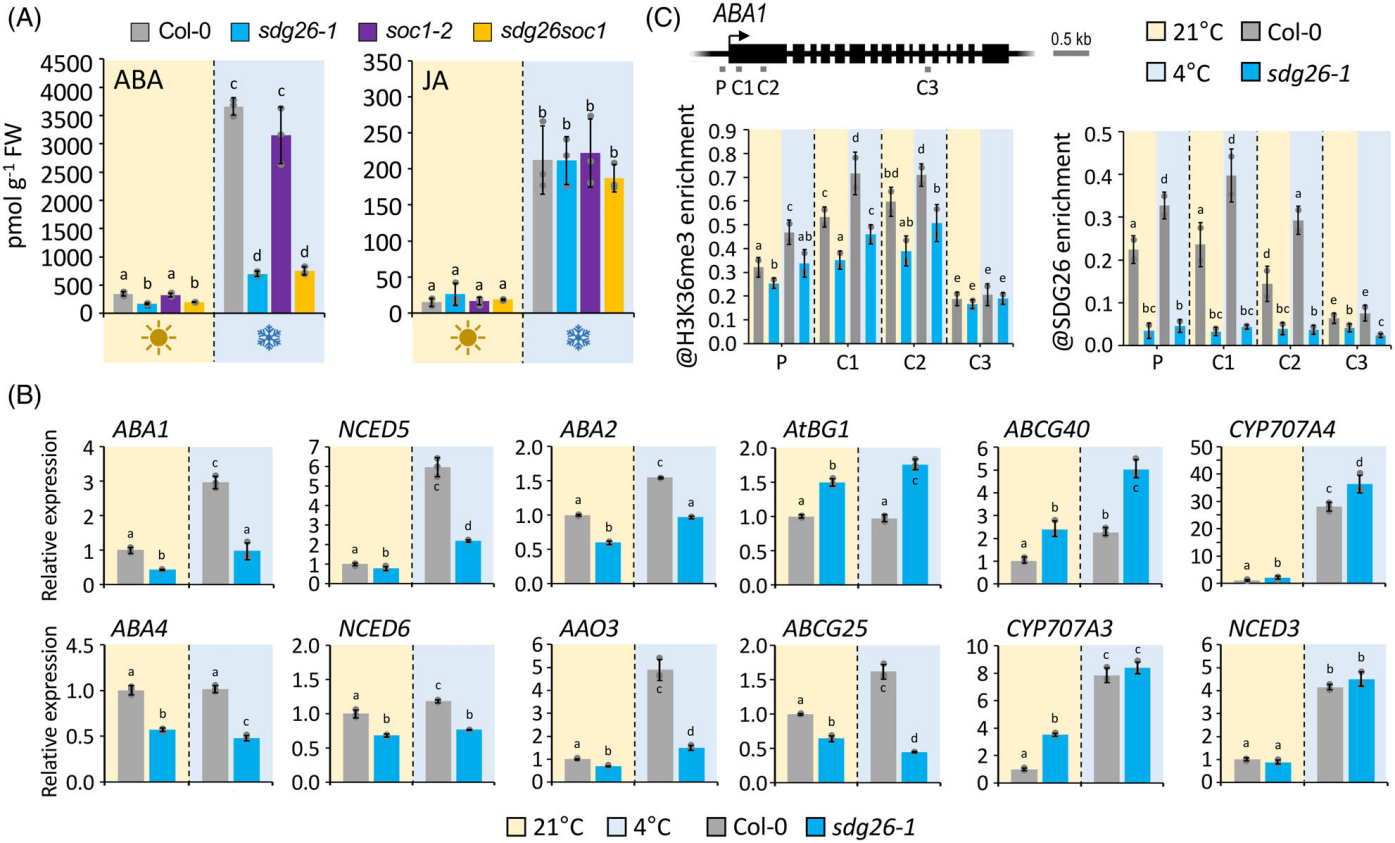
ABA homeostasis in response to cold. (A) ABA (left) and JA (right) levels were quantified in 2‐week‐old Col‐0, *sdg26‐1*, *soc1‐2*, and *sdg26soc1* seedlings at 21°C or after cold stress at 4°C. Letters indicate significant differences (*P* < 0.05). (B) Expression analyses of key genes involved in regulation of ABA homeostasis in 2‐week‐old Col‐0 and *sdg26‐1* plantlets treated with a cold stress for 4 h at 4°C or not (RT). Data are expressed relative to Col‐0 level at RT (set as 1) as mean ± SD of at least three biological replicates. (C) The levels of H3K36me3 and SDG26 along different regions of *ABA1* were measured by ChIP in 2‐week‐old plantlets treated with a cold stress for 4 h at 4°C or not (RT). Data are presented as the ratio of H3K36me3 and SDG26 over H3 for each amplified region. Mean values ± SD are presented based on results from two biological replicates. Letters indicate significant differences (Student's *t*‐test with Benjamini–Hochberg FDR correction, *P* < 0.05).

The regulation of ABA biosynthesis, metabolism, and transport is critical for adjusting ABA levels in response to abiotic stress (Baron et al., 2012). To uncover the underlying mechanisms, we reexamined transcriptomic data from *sdg26‐1* grown under normal condition (Liu, Berr, et al., 2016) and identified several misregulated genes involved in ABA homeostasis (Table S1). We subsequently examined by qRT‐PCR a comprehensive set of ABA biosynthesis, catabolism, transport, and conjugation genes described by Baron et al. (2012). While several tested genes, including *NCED2, NCED3, NCED9, CYP707A1, CYP707A2*, and *UGT71B6*, were not significantly altered in *sdg26‐1* under either control or cold conditions (exemplified for *NCED3* in Figure 4B), key ABA biosynthesis genes (*ABA1*, *ABA4*, *NCED5*, *NCED6*, *ABA2*, and *AAO3*) were downregulated in *sdg26‐1* (Figure 4B). In line with this, the ABA exporter *ABCG25* was also reduced, whereas the ABA importer *ABCG40* and ABA‐specific β‐glucosidase gene *AtBG1* were upregulated. Consistent with the importance of a precise intracellular ABA homeostasis (Baron et al., 2012), the ABA catabolic genes *CYP707A3* and *CYP707A4* (i.e., which belong to the CYP707A subfamily, a key regulator of ABA degradation) were upregulated, particularly under non‐stress conditions. Importantly, these misregulations were restored in the p+gSDG26‐Myc line (Supplementary Figure S7), confirming direct and/or indirect SDG26‐dependent control. Chromatin analysis provided direct evidence for this regulation at the *ABA1* locus. Indeed, H3K36me3 was lower in *sdg26‐1* compared to Col‐0, both at control and cold conditions, and SDG26 binding was detected at the 5’ region of the gene (Figure 4C). Together, these data demonstrate that *SDG26* influences ABA homeostasis by transcriptionally regulating several genes involved in ABA biosynthesis, transport, and degradation.

ABA is a key hormone in stress adaptation (Vishwakarma et al., 2017). For example, it promotes stomatal closure under stress conditions, including low temperatures, thereby limiting leaf dehydration (Agurla et al., 2018). Given that *sdg26‐1* accumulates less ABA than wild‐type plants, we next assessed typical ABA‐dependent physiological responses. At 21°C, the proportions of open, partially open and closed stomata were comparable among Col‐0, *sdg26‐1*, *soc1‐2*, and *sdg26soc1*. Under cold, *sdg26‐1* and *sdg26soc1* exhibited significantly fewer closed stomata than Col‐0, indicating impaired cold‐induced stomatal closure (Figure 5A). Besides this, no differences in other stomatal parameters (i.e., density, index size) were detected between the different analyzed genotypes (Figure S9). ABA is also a central regulator of plant adaptation to drought. Consistent with reduced ABA, *sdg26‐1* and *sdg26soc1* plants were also more sensitive to drought stress, displaying lower survival after water deprivation and faster water loss from detached leaves (Figure 5B,C). Finally, ABA also regulates seed dormancy and germination. While all genotypes germinated similarly after stratification, *sdg26‐1* and *sdg26soc1* seeds germinated more rapidly than Col‐0 and *soc1‐2* without stratification (Figure 5D,E). This reduced dormancy suggests that ABA deficiency in *sdg26‐1* may also extend to the embryonic stage. Finally, because loss of *SOC1* function was previously shown to enhance freezing tolerance (Seo et al., 2009), we also exposed *sdg26‐1* to a freezing treatment. Interestingly, despite opposite effect on CBF‐COR genes (Figure 3A), *sdg26‐1* displayed survival rates after freezing that were statistically indistinguishable from *soc1‐2* and significantly higher than Col‐0 (Figure S10; *P* = 0.0953). Taken together, these findings highlight *SDG26* as an important regulator for ABA homeostasis, which in turn modulates stress‐responsive traits such as stomatal closure, drought tolerance, and seed dormancy. Although, despite compromising both *CBF‐COR* induction and ABA homeostasis, loss of *SDG26* paradoxically improves freezing tolerance, pointing to the involvement of additional, CBF‐ and ABA‐independent mechanisms that remain to be determined.

**Figure 5:**
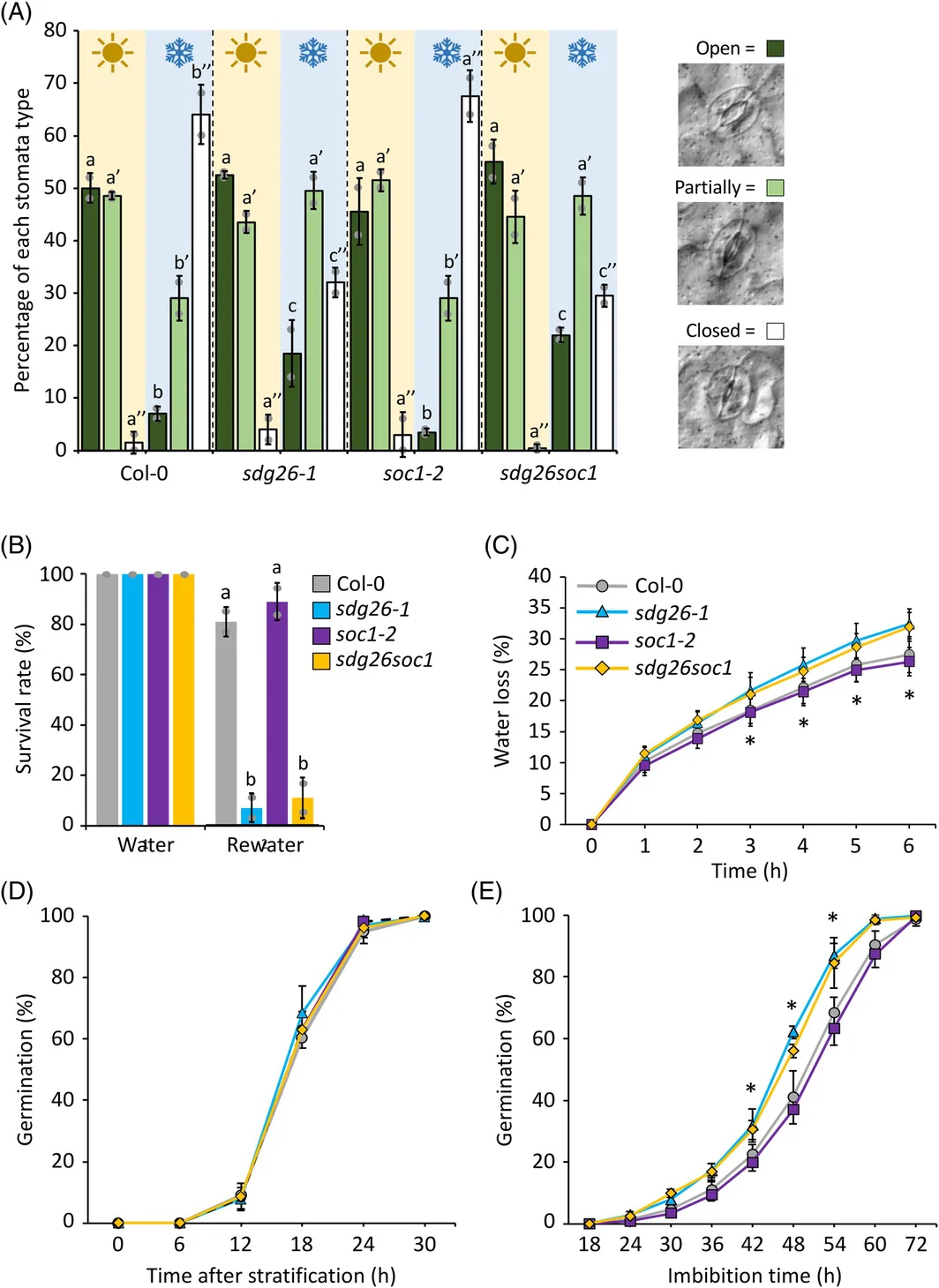
ABA‐related phenotypes under cold, drought, and germination conditions. (A) Stomatal closure was assessed by counting stomata in three categories (Open, Partially open, and Closed) before (21°C) and after 1 h of cold stress (4°C). The experiment was repeated twice with similar results. (B) Survival rates were recorded after withholding water for 10 days followed by 3 days of rewatering. Data represent mean ± SD of 20 plants per genotype. The experiment was repeated three times with similar outcomes. Letters indicate significant differences (Student's *t*‐test with Benjamini–Hochberg FDR correction, *P* < 0.05). (C) Water loss was quantified hourly over 6 h using five detached leaves from three plants per genotype. Data represent mean ± SD from a representative experiment. Two additional biological replicates produced similar results. Germination rates of Col‐0, *sdg26‐1*, *soc1‐2*, and *sdg26soc1* seeds with (D) or without (E) stratification. Germination was scored based on radicle emergence. Data represent mean ± SD from five independent experiments with 100 seeds per genotype per replicate. Asterisks in (C) and (E) indicate significant differences between *sdg26‐1* and Col‐0 (*P* < 0.05).

## DISCUSSION

In this study, we comprehensively characterize the histone methyltransferase *SDG26*, and demonstrate its key role in Arabidopsis responses to abiotic stress, particularly cold stress. SDG26 was originally classified as a TrxG histone methyltransferase due to its SET domain and phylogenetic similarity to ASH1‐type enzymes (Baumbusch et al., 2001). Like other TrxG proteins (Zhao et al., 2005) (Puig et al., 2007) (Berr et al., 2009) (Chen et al., 2017), SDG26 localizes to the nucleus and is broadly expressed across organs, with particularly strong activity in vascular tissues (Figures S1, S2). *In situ* hybridization revealed transcript enrichment in young tissues and reproductive structures such as anthers and ovules (Figure S2), echoing the expression domains of *SDG8* and *SDG2* (Zhao et al., 2005) (Berr, McCallum, Ménard, et al., 2010). Promoter‐GUS analyses revealed both overlapping and distinct expression domains among TrxG proteins. Similar to *ATX1‐5* (Saleh et al., 2007) (Hayot et al., 2012) (Chen et al., 2017), the *SDG26* promoter drives strong GUS activity in vascular tissues (Figure S1), a pattern absent from *SDG7* and *SDG8* promoters (Kim et al., 2005) (Berr, McCallum, Alioua, et al., 2010) (Lee et al., 2015). In root tips, GUS staining was detected for *ATX1, ATX2, SDG7, SDG8*, and *SDG26* (Saleh et al., 2007) (Berr, McCallum, Alioua, et al., 2010) (Lee et al., 2015) (Figure S1), but not for *ATX3‐5* (Chen et al., 2017). Likewise, GUS activity in flowers is broadly shared among *SDG26*, *SDG8*, and *ATX1‐5* (Saleh et al., 2007) (Cazzonelli et al., 2009) (Hayot et al., 2012) (Chen et al., 2017) (Figure S1D). Notable differences have also been reported, including *SDG8* activity in hydathodes (Berr, McCallum, Alioua, et al., 2010) and *ATX3‐5* expression in trichomes (Chen et al., 2017). Collectively, these observations show that TrxG genes display broad but also partially overlapping expression consistent with the physical interactions already documented between SDG26 and ATX1 or SDG8 (Valencia‐Morales et al., 2012). In addition, these results suggest that, in connection or not with its function in flowering‐time regulation, SDG26 is likely involved in additional developmental processes.

In this work, we demonstrated that SDG26 is induced by abiotic stresses but not by stress‐related phytohormones (Figure S5), a regulation confirmed at both transcript and protein levels (Figure 1) and apparently conserved, since the SDG26 ortholog in *Populus trichocarpa* is also strongly upregulated in dormant winter stems (Ko et al., 2011). Although transcript and protein levels were stress‐responsive, SDG26 nuclear localization remained stable, with no evidence for long‐distance mobility or altered subnuclear distribution under stress (Figures S4, S6). Previously, we established that SDG26 regulates *SOC1* during flowering (Berr et al., 2015) (Liu, Berr, et al., 2016) and *SOC1* itself was proposed to mediate crosstalk between cold sensing and flowering (Seo et al., 2009). Here, we observed that *SOC1* was rapidly induced by cold in Col‐0 plants (Figure 2A), whereas its induction was reduced in *sdg26* and correlated with lower enrichment of H3K4me3 and H3K36me3 at *SOC1* chromatin (Figure 2B). Despite this induction, SDG26 occupancy at *SOC1* chromatin in Col‐0 remained stable during cold treatment, suggesting that its contribution to *SOC1* activation does not involve dynamic recruitment but rather the maintenance of permissive chromatin states (Figure 2B). Seo et al. (2009) proposed that *SOC1* represses *CBF‐COR* genes. Yet, despite reduced *SOC1* induction in *sdg26*, *CBF‐COR* genes were also less induced by cold (Figure 3A). This result is consistent with our finding that SDG26 directly binds *CBF* loci, where its occupancy and associated H3K36me3 enrichment increase after cold exposure but are reduced in *sdg26* (Figure 3B). Together these findings indicate that SDG26 contributes to cold responses not only through *SOC1* but also via activation of *CBF* genes. This dual role raises the possibility that SOC1 and SDG26 may compete or cooperate for binding at *CBF*, possibly through shared cis‐regulatory elements. In addition, since CBF expression is modulated by transcription factors such as ICE1/2, CAMTA1–3, PIF4/7, and MYB15 (Agarwal et al., 2006; Doherty et al., 2009; Lee & Thomashow, 2012), SDG26 recruitment may also be coordinated with these factors. Whether SDG26 acts alone or in combination with such regulators remains an open question.

Beyond its role in *SOC1* and *CBF* regulation, our results show that SDG26 also contributes to cold responses through ABA‐dependent pathways by maintaining ABA homeostasis (Figure 4), thereby supporting ABA‐mediated processes such as stomatal regulation, drought tolerance, and seed dormancy. Among the downregulated genes in *sdg26*, *ABA1*, and *ABA2* are known to promote flowering (Riboni et al., 2016), suggesting that the late‐flowering phenotype of *sdg26* may partly result from reduced endogenous ABA. Interestingly, the rice homolog of *SDG26*, *SDG708*, also promotes flowering, and its loss of function results in delayed flowering similar to Arabidopsis *sdg26* mutants (Sui et al., 2013), pointing to a conserved role of SDG26‐like proteins in reproductive timing. Since ABA promotes early flowering in rice (Du et al., 2018), it will be important to determine whether SDG708, like Arabidopsis SDG26, also contributes to ABA homeostasis in a conserved mechanism linking chromatin regulation, ABA signaling, and flowering across species. Additional pathways may further connect ABA signaling to *SOC1* regulation. Under drought stress, ABF3 and ABF4 together with NF‐YCs were shown to promote *SOC1* transcription, thereby accelerating flowering and facilitating life cycle completion under stress (Hwang et al., 2019). This raises the possibility that SDG26 influences flowering and stress adaptation both through direct chromatin regulation of *SOC1* and indirectly by modulating ABA levels, which could feed into ABA‐dependent *SOC1* induction under specific stress conditions. Finally, ABA itself has long been recognized as a central regulator of cold acclimation (Shi et al., 2015), reinforcing the view that SDG26 integrates ABA‐dependent and ABA‐independent pathways to fine‐tune the balance between stress resilience and reproductive development. Thus, SDG26 acts as a critical hub integrating flowering regulation, ABA signaling, and abiotic stress response. Its known role in activating *SOC1* and modulating flowering time, combined with its effect on cold responding genes induction, ABA levels and stress tolerance, supports a model in which SDG26 orchestrates a coherent developmental and physiological program. The combination of late flowering, reduced ABA accumulation, impaired stomatal dynamics, enhanced drought susceptibility and freezing tolerance, observed in *sdg26* plants is consistent with ABA's dual role in promoting both stress resilience and reproductive transition (Jiang et al., 2025). As such, SDG26 may help balance these processes under adverse conditions, allowing plants to optimize survival while ensuring timely flowering, and potentially promoting flowering as a strategy to complete the life cycle under stress. This dual role is not unprecedented. ATX1 regulates drought responses via an ABA‐dependent pathway (Ding et al., 2011), and protein–protein interactions between ATX1 and SDG26 have been reported (Valencia‐Morales et al., 2012), raising the possibility of cooperative regulation of ABA‐related stress responses. Similarly, SDG8 and ATX1 are also known to regulate both flowering and stress pathways (Ramirez‐Prado et al., 2018). Such integrated roles may provide an adaptive advantage by coordinating development and stress acclimation to maximize survival and reproductive success under fluctuating environments.

Finally, functional studies have shown that SDG26 promotes H3K4 and H3K36 trimethylation at target loci such as *SOC1*, thereby impacting transcription (Berr et al., 2015; Liu, Berr, et al., 2016). More recent evidence indicates that SDG26 can also participate in repressive complexes, as in the case of *FLC* regulation where it associates with RNA‐processing factors and influences H3K27me3 states (Fang et al., 2020; Qi et al., 2022). Our results extend this view by showing that SDG26 directly binds cold‐responsive loci, including *CBF* genes and *ABA1*, and that its loss reduces H3K36me3 enrichment at these sites, underscoring its versatile role in gene regulation. This versatility reflects a broader paradigm in plant chromatin, where histone methylation, and in particular H3K36, acts as a central regulator of stress responses by shaping both acute transcriptional reprogramming and transcriptional memory (Yu et al., 2025). A recent study showed that SDG26 plays a context‐dependent and cooperative role in fine‐tuning chromatin states by contributing to H3K36me3 deposition. Together with SDG8 and SDG25, it helps establish locus‐specific H3K36me3 landscapes, including at long, stress‐responsive genes, thereby contributing to the formation of a “transcription‐resistive” chromatin state, an open but transcriptionally inert configuration associated with transcript stabilization (Yao et al., 2025). Within this framework, SDG26 emerges as a context‐dependent and cooperative factor that fine‐tunes chromatin states at selected genes, thereby integrating developmental and environmental cues into adaptive transcriptional programs.

## MATERIALS AND METHODS

### Plant materials

All *Arabidopsis thaliana* lines used in this study were in the Columbia ecotype (Col‐0) background. Col‐0 is referred to as the wild‐type (WT) plant. The mutants *sdg26‐1* (Xu et al., 2008) and *soc1‐2* (Lee et al., 2000) were previously described. The *sdg26 soc1* double mutant and all the transgenic lines (*pSDG26‐GUS*, *p+gSDG26‐myc*, and *p35S+gSDG26‐GFP*) mentioned herein after were generated through genetic crosses or Agrobacterium‐mediated transformation. Homozygotes were characterized by antibiotic resistance selection and/or PCR‐based genotyping using specific primers (Table S2).

### Generation of transgenic lines

The 1772 bp *SDG26* promoter region, the *SDG26* gene along with its promoter region, and the SDG26 CDS were separately PCR amplified using specific primer pairs (Table S2). These fragments were cloned into the Gateway donor vector pDONR207 and then subcloned into the binary vectors pGWB633, pGWB19, and pB7FWG2.0. The resulting constructs (i.e., *pSDG26‐GUS*, *p+gSDG26‐MYC*, and *p35S+gSDG26‐GFP*) were introduced into *Agrobacterium tumefaciens* GV3101, and transformation was carried out by floral dip. Homozygous T2 transgenic Arabidopsis plants were selected using antibiotic resistance.

### *In situ* hybridization and GUS staining

Digoxigenin labeling of RNA probes, tissue preparation, and *in situ* hybridization were performed as previously described (Berr *et al*., 2010). Tissue sections were 8 μm thick. A 214 bp fragment covering the 3′‐end and the 3′‐UTR of *SDG26* was obtained through PCR amplification using specific primers (see Table S2). This fragment was cloned into the pGEM‐T Easy vector (Promega) and used for the preparation of sense and antisense probes. For GUS expression analysis via histochemical staining, the T2 progeny from six independent homozygous primary transformants were examined as previously described (Berr, McCallum, Alioua, et al., 2010; Berr, McCallum, Ménard, et al., 2010).

### Plant micrografting

Micrografting between the hypocotyls of rootstocks and scions was carried out without collars on 6‐day‐old seedlings, as previously described (Regnault et al., 2015). Successful grafts were then transferred to MS medium for fluorescence microscopic observation or to soil for flowering time analyses.

### Stress treatments

Plants grown for 2 weeks on Murashige and Skoog (MS) medium in a growth chamber under long‐day (LD; 16 h light: 8 h dark) photoperiod at 21°C were subjected to various types of stress. For chemical treatments, *in vitro*‐grown plants were transferred to liquid MS medium supplemented with 100 mM NaCl, 10 μM ABA (Sigma‐Aldrich, A1049), 10 μM ACC (Sigma‐Aldrich, A3903), 1 μM Kinetin (Sigma‐Aldrich, K3378), 1mM SA (Sigma‐Aldrich, S5922), or 10 μM MeJA (Sigma‐Aldrich, 392707). For cold stress, plants were transferred to 4°C or maintained at 21°C for 0, 4, 6, 8, 12 or 16h in the light, following the procedure described in Seo et al. (2009). The zero time point corresponds to right after dawn and all treatments were performed under the same light conditions as control plants. For drought stress, plants grown in soil under medium‐day (MD; 12 h light: 12 h dark) photoperiod at 21°C for 10 days were either watered or left unwatered. After a week, stressed plants were rewatered for 3 days, and survival rates were recorded. In parallel, water loss was measured using detached leaves from 2‐week‐old non‐stressed plants by weighting at different time points. Finally, freezing tolerance was assessed in plants grown for 2 weeks in soil at 21°C by exposing them to –10°C. After 2 h, the plants were allowed to recover in a growth chamber at 21°C, and survival rates were evaluated after 1 week.

### Germination tests

Seeds were surface‐sterilized 5 min in 75% ethanol with 0.01% (v/v) Triton‐X 100, rinsed with sterile distilled water and distributed on the surface of ½ MS plates in Petri dishes. Germination was assessed by counting the visible radicle emergence of seeds subjected or not to cold pretreatment (i.e., stratification for 48 h at 4°C in the dark). Five biological replicates consisting of 100 seeds per replicate were used for each experiment.

### Hormones quantification

ABA and jasmonate were quantified using UPLC‐MS/MS, as described previously (Smirnova et al., 2017). Briefly, Col‐0, *sdg26‐1*, *soc1‐2*, and *sdg26soc1* plants were grown on MS for 2 weeks under LD conditions and subjected to cold stress at 4°C for 8 h or maintained at 21°C. Approximately 100 mg of fresh plant material from each genotype was collected, ground in an extraction solution (6xV/W; 70% methanol, 29% water, 1% acetic acid) containing internal standard (deuterated ABA or dABA), and immediately frozen in liquid nitrogen. The extraction solutions were analyzed using the following MRM transitions: JA (209 > 59, –), JA‐Ile (324 > 151, +), ABA (263 > 153, –), and dABA (269 > 159, –), with + and – indicating analysis in positive or negative mode, respectively. Quantitative profiles were obtained using an EVOQ Elite LC‐TQ (Bruker) equipped with an electrospray ionization source and coupled to a Dionex UltiMate 3000 UHPLC system (Thermo). Data acquisition was performed with the MS Workstation 8 for mass spectrometry, while liquid chromatography was controlled by Bruker Compass Hystar 4.1 SR1 software. Data analysis was conducted using the MS Data Review software. Absolute quantifications were achieved by comparing sample signals to calibration curves established with pure compounds (ABA: 2–1000 nM; JA: 2–1000 nM), using a linear regression through the origin (*R* ^2^ >0.996). Sample areas were normalized to the deuterated internal standard signal and corrected for fresh weight. The final hormone concentrations are expressed in pmol g^−1^ fresh weight (FW), calculated using the formula: Concentration (pmol g^−1^ FW) = (Slope × Aire_brut_) × (IS_ref_/Aire_SI_) × 20, where Slope is the calibration curve slope, Aire_brut_ is the sample peak area, IS_ref_ is the mean internal standard area across all samples, Aire_SI_ is the sample‐specific internal standard area, and the factor 20 accounts for the extraction volume (100 μL), injection volume (5 μL), and sample fresh weight (100 mg).

### Stomata analysis

Epidermal strips were collected from plants maintained at 21°C or subjected to 1h of cold stress at 4°C and fixed in a solution containing 1% glutaraldehyde, 10 mM PIPES (pH 7.0), 5 mM MgCl_2_, and 5 mM EGTA for 1 h. The samples were then cleared overnight in chloral hydrate, and stomata were examined under a Nikon Eclipse 800 microscope. Microscopy images were captured using a Hitachi S‐3400N (Hitachi High‐Technologies Europe). All images were processed using ImageJ (NIH).

### Western blotting

Western blot analysis was performed on total protein extracts prepared from 2‐week‐old seedlings, as described previously (Zhang et al., 2020). Protein samples were separated on 8% SDS‐PAGE and transferred onto Immobilon‐P PVDF membranes (Merck Millipore). The membranes were probed with antibodies against GFP (11120; Sigma‐Aldrich), MYC (C3956; Sigma‐Aldrich), or H3 (ab12079; Abcam), followed by a goat anti‐mouse or anti‐rabbit HRP‐conjugated secondary antibodies (62‐6520, Thermofisher; A9169, Sigma‐Aldrich). Chemiluminescence was detected with ECL+ (Lumi‐Light PLUS Western Blotting; Roche) and visualized with ECL films (Bio‐Rad). The intensity of individual bands was quantified using ImageJ (NIH).

### Gene expression analyses by qPCR

Total RNA was isolated using the Nucleospin RNA Plant Kit (Macherey‐Nagel). First‐strand cDNA was synthesized using SuperScript® IV Reverse Transcriptase (Invitrogen) with an oligo‐dT primer, following the manufacturer's recommendations. Transcript abundance was quantified in triplicates using gene‐specific primers (Table S2) in a 10 μL reaction mix containing Takyon™ No Rox SYBR® MasterMix Blue dTTP on a LightCycler 480 (Roche). *EXP* (AT4G26410) and *TIP4.1* (AT4G34270) were used for normalization. Relative expression levels were calculated using the 2^−ΔΔCT^ method.

### Histone modifications analyses

Chromatin immunoprecipitation was performed as previously described (Liu, Berr, et al., 2016). Chromatin was precipitated using anti‐H3 (ab12079; Abcam), anti‐trimethyl‐H3K36 (ab9050; Abcam), anti‐MYC (C3956; Sigma‐Aldrich), anti‐GFP (ab290; Abcam), or anti‐SDG26 (Berr et al., 2015), along with protein A magnetic beads (Magna‐ChIP, Millipore). After immunoprecipitation, DNA was recovered using the NucleoSpin Gel and PCR Clean‐up kit (Macherey‐Nagel) and analyzed by real‐time PCR (LightCycler 480II; Roche) in conjunction with the SYBR Green Master mix using gene‐specific primers (Table S2).

### Statistical methods

Statistical analyses were performed in R (http://www.r‐project.org/) using Student's *t*‐tests with Benjamini–Hochberg false discovery rate (FDR) correction.

## Author Contributions

AB initiated and coordinated the project. AB and XZ designed the experiments. XZ, JL, JZ, ME, and AB performed experiments. AB and XZ made figures and wrote the manuscript. JL, JZ, ME, and W‐HS revised the manuscript. All authors approved the final version of the manuscript.

## Conflict of Interest

The authors declare no competing interests.

## Supporting information


**Figure S1.** GUS staining in transgenic plants harboring a GUS reporter gene driven by the SDG26 promoter. (A) Mature (left) and cauline (right) leaves from 6‐week‐old plants. (B) Siliques at different developmental stages, 3–4 Days Post Anthesis (DPA; left) and 9–11 DPA. (C) Base of shoot branches and cauline leaf petiole. (D) Total inflorescence and different flower details, from young flower buds (top left), young flower (top middle), open flower (top right), stigma after pollination (bottom left), young silique (bottom middle), and anther (bottom right). (E) Root tip with the root cap (RC) and the meristematic zone (MZ; left) and maturation zone with emerging lateral root (LR; right). (F) A 15‐day‐old seedling. (G) Close‐up of the lateral leaf axillary meristem (LAM) of a 10‐day‐old seedling. (H) Cotyledon of a 10‐day‐old seedling.

**Figure S2.** Expression pattern analysis of *SDG26*. (A–G) *In situ* hybridization analysis of *SDG26* in: (A) a floral apex with (fl) for flower primordia and (am) for apical meristem; (B) a vegetative apex with the Shoot Apical Meristem (SAM) in the center; (C) an embryo at mid‐torpedo stage; (D, E) anthers at stage 9 with (ms) for microspore; (F) a gynoecium with (o) for ovule; and (G) a leaf dissected from a 2‐week‐old seedling with at the center the mid‐vein. Negative controls using a sense probe did not generate detectable signal. Bars = 100 μm.

**Figure S3.** Analyses of *sdg26‐1* transgenic plants expressing p35S‐gSDG26‐GFP or p + gSDG26‐Myc. (A) The expression of each transgenic SDG26 protein was detected in indicated transgenic lines by western blot using either an anti‐GFP or anti‐c‐Myc antibodies. The *sdg26‐1* mutant was used as a negative control. (B) The transcript level of *SDG26* was quantified by qPCR in different transgenic lines. (C) Measures of flowering time, either as the number of rosette leaves or the number of days at bolting, of each transgenic line are presented as mean ± SD of two biological replicates (*n* > 15 individual plants). (D) Transcript levels of flowering genes affected in *sdg26‐1* were quantified in *sdg26* transgenic lines. Expression analyses in (B) and (D) were determined in 2‐week‐old plantlets grown under MD and are presented relative to the Col‐0 level (set as 1). Letters in (B), (C), and (D) indicate significant differences (Student's *t*‐test with Benjamini–Hochberg FDR correction, *P* < 0.05). Confocal images of transgenic root (E), transgenic leaves (F), transgenic stomata cells (G), and transgenic pavement cells (H). Scale bar represents 100 μm in (E) and (F) or 25 μm in (G) and (H).

**Figure S4.** Analyses of the SDG26 protein mobility by different grafting combinations on *sdg26‐1* mutant plants. (A) The flowering phenotypes of Col‐0 scions grafted on *sdg26‐1* rootstocks (Col‐0/*sdg26*) and vice versa (*sdg26*/Col‐0) were compared to Col‐0 scions grafted on Col‐0 rootstock (Col‐0/Col‐0) and *sdg26‐1* scions grafted on s*dg26‐1* rootstocks (*sdg26*/*sdg26*). Ten days after grafting, successfully grafted plants were transferred to soil and the flowering time was latter measured from each grafting combination grown under mid‐day conditions (16 h light/8 h dark). Representative plants of each of the four grafting combinations are presented with emerging inflorescence indicated with a white arrow. Letters indicate significant differences (Student's *t*‐test with Benjamini–Hochberg FDR correction, *P* < 0.05). (B) Detection of SDG26‐GFP fluorescent signals by confocal microscopy in leaf (left) or root (right) from *sdg26‐1* scion grafted on *sdg26‐1* p35S‐SDG26‐GFP transgenic rootstock (top) or *sdg26‐1* p35S‐SDG26‐GFP scion grafted on *sdg26‐1* rootstock. Scale bar represents 100 μm.

**Figure S5.** Analyses of the SDG26 expression in response to stress‐related phytohormones. (A–E) The *SDG26* transcript level was quantified in 2‐week‐old plantlets Col‐0 plants grown at 21°C under LD conditions and treated with different stress‐related‐phytohormones (A) ABA, (B) ACC, (C) Kinetin, (D) SA, or (E) MeJA. For each condition, a reference gene known to be transcriptionally induced was used as a control. The expression levels of each gene are presented relative to their level before treatment (set as 1) as box plots. Letters indicate significant differences (Student's *t*‐test with Benjamini–Hochberg FDR correction, *P* < 0.05).

**Figure S6.** Cellular localization of SDG26 in stressed plants. Using confocal microscopy, the nuclear localization of the florescent SDG26‐GFP protein was compared between *sdg26‐1* transgenic plants grown at 21°C or treated for 4 h at 4°C.

**Figure S7.** Analyses of the expression of different cold‐induced genes in wild‐type Col‐0 and *sdg26‐1* transgenic plants expressing *p + gSDG26‐Myc*. Transcript levels were quantified in 2‐week‐old plantlets grown on MS medium under MD photoperiod conditions before (0 h) and during (4 h) a cold treatment at 4°C. The expression levels of each gene are presented relative to their level in Col‐0 at 0 h (set as 1). Letters indicate significant differences (*P* < 0.05).

**Figure S8.** Transcript analysis of cold‐inducible genes under resting conditions. (A) Expression analyses of cold‐inducible genes in 2‐week‐old Col‐0, *sdg26‐1*, *soc1‐2*, and *sdg26soc1* plantlets grown at room temperature in parallel to plants treated by cold presented in Figure 3A. Data are presented relative to Col‐0 at 0 h.

**Figure S9.** Stomatal characteristics in wild‐type Col‐0 plants and *sdg26‐1*, *soc1‐2*, and *sdg26soc1* mutants. Representative scanning electron microscopy images of stomata from wild‐type Col‐0 and *sdg26‐1*, *soc1‐2*, and *sdg26soc1* mutant plants grown on MS under MD photoperiod conditions. Bars = 5 μm. Stomatal density (the number of stomata per area), stomatal index (the number of stomata per total epidermal cells), stomata size were analyzed in the leaf abaxial epidermal layers of the different indicated genotypes.

**Figure S10.** Freezing tolerance of wild‐type Col‐0 plants and *sdg26‐1*, *soc1‐2*, *sdg26soc1*, and *sdg8‐1* mutants. (A) and (B) Representative pictures showing plants before freezing treatment (21°C) and freeze‐treated plants after recovery (−10°C). The percentage of plants that survived after freezing (survival rate) was calculated by counting the number of living plants before freezing treatment and after recovery. Letters indicate significant differences (*P* < 0.05).

**Table S1.** Genes involved in ABA homeostasis according to Baron et al. (2012) and mis‐regulated in sdg26‐1 under normal growth conditions based on microarray data from Liu, Berr, et al., (2016).

**Table S2.** Primers used in this work.

## Data Availability

The data supporting this work are available within the paper and its Supporting Information.

## References

[tpj71180-bib-0001] Agarwal, M. , Hao, Y. , Kapoor, A. , Dong, C.‐H. , Fujii, H. , Zheng, X. et al. (2006) A R2R3 type MYB transcription factor is involved in the cold regulation of CBF genes and in acquired freezing tolerance. The Journal of Biological Chemistry, 281, 37636–37645. 10.1074/jbc.M605895200 17015446

[tpj71180-bib-0002] Agurla, S. , Gahir, S. , Munemasa, S. , Murata, Y. & Raghavendra, A.S. (2018) Mechanism of Stomatal Closure in Plants Exposed to Drought and Cold Stress. In: Iwaya‐Inoue, M. , Sakurai, M. & Uemura, M. (Eds.) Survival Strategies in Extreme Cold and Desiccation: Adaptation Mechanisms and Their Applications. Advances in Experimental Medicine and Biology. Singapore: Springer, pp. 215–232. Available from: 10.1007/978-981-13-1244-1_12 30288712

[tpj71180-bib-0003] Barnaby, J.Y. , Kim, J. , Devi, M.J. , Fleisher, D.H. , Tucker, M.L. , Reddy, V.R. et al. (2020) Varying atmospheric CO_2_ mediates the cold‐induced CBF‐dependent signaling pathway and freezing tolerance in Arabidopsis. International Journal of Molecular Sciences, 21, 7616. 10.3390/ijms21207616 PMC7593905 33076265

[tpj71180-bib-0004] Baron, K.N. , Schroeder, D.F. & Stasolla, C. (2012) Transcriptional response of abscisic acid (ABA) metabolism and transport to cold and heat stress applied at the reproductive stage of development in Arabidopsis thaliana. Plant Science, 188–189, 48–59. 10.1016/j.plantsci.2012.03.001 22525244

[tpj71180-bib-0005] Baumbusch, L.O. , Thorstensen, T. , Krauss, V. , Fischer, A. , Naumann, K. , Assalkhou, R. et al. (2001) The Arabidopsis thaliana genome contains at least 29 active genes encoding SET domain proteins that can be assigned to four evolutionarily conserved classes. Nucleic Acids Research, 29, 4319–4333. 10.1093/nar/29.21.4319 PMC60187 11691919

[tpj71180-bib-0006] Berr, A. , McCallum, E.J. , Alioua, A. , Heintz, D. , Heitz, T. & Shen, W.‐H. (2010) Arabidopsis histone methyltransferase SET DOMAIN GROUP8 mediates induction of the Jasmonate/ethylene pathway genes in plant defense response to necrotrophic fungi. Plant Physiology, 154, 1403–1414. 10.1104/pp.110.161497 PMC2971616 20810545

[tpj71180-bib-0007] Berr, A. , McCallum, E.J. , Ménard, R. , Meyer, D. , Fuchs, J. , Dong, A. et al. (2010) Arabidopsis SET DOMAIN GROUP2 is required for H3K4 trimethylation and is crucial for both sporophyte and gametophyte development. Plant Cell, 22, 3232–3248. 10.1105/tpc.110.079962 PMC2990135 21037105

[tpj71180-bib-0008] Berr, A. , Shafiq, S. , Pinon, V. , Dong, A. & Shen, W.‐H. (2015) The trxG family histone methyltransferase SET DOMAIN GROUP 26 promotes flowering via a distinctive genetic pathway. The Plant Journal, 81, 316–328. 10.1111/tpj.12729 25409787

[tpj71180-bib-0009] Berr, A. , Xu, L. , Gao, J. , Cognat, V. , Steinmetz, A. , Dong, A. et al. (2009) SET DOMAIN GROUP25 encodes a histone methyltransferase and is involved in FLOWERING LOCUS C activation and repression of flowering. Plant Physiology, 151, 1476–1485. 10.1104/pp.109.143941 PMC2773071 19726574

[tpj71180-bib-0010] Campi, M. , D'Andrea, L. , Emiliani, J. & Casati, P. (2012) Participation of chromatin‐remodeling proteins in the repair of ultraviolet‐B‐damaged DNA. Plant Physiology, 158, 981–995. 10.1104/pp.111.191452 PMC3271783 22170978

[tpj71180-bib-0011] Cazzonelli, C.I. , Yin, K. & Pogson, B.J. (2009) Potential implications for epigenetic regulation of carotenoid biosynthesis during root and shoot development. Plant Signaling & Behavior, 4, 339–341. 10.4161/psb.4.4.8193 PMC2664501 19794857

[tpj71180-bib-0012] Chen, L.‐Q. , Luo, J.‐H. , Cui, Z.‐H. , Xue, M. , Wang, L. , Zhang, X.‐Y. et al. (2017) ATX3, ATX4, and ATX5 encode putative H3K4 methyltransferases and are critical for plant development. Plant Physiology, 174, 1795–1806. 10.1104/pp.16.01944 PMC5490889 28550207

[tpj71180-bib-0013] Ding, Y. , Avramova, Z. & Fromm, M. (2011) The Arabidopsis trithorax‐like factor ATX1 functions in dehydration stress responses via ABA‐dependent and ABA‐independent pathways. The Plant Journal, 66, 735–744. 10.1111/j.1365-313X.2011.04534.x 21309869

[tpj71180-bib-0014] Doherty, C.J. , Buskirk, H.A.V. , Myers, S.J. & Thomashow, M.F. (2009) Roles for Arabidopsis CAMTA transcription factors in cold‐regulated gene expression and freezing tolerance. The Plant Cell, 21, 972–984. 10.1105/tpc.108.063958 PMC2671710 19270186

[tpj71180-bib-0015] Du, H. , Huang, F. , Wu, N. , Li, X. , Hu, H. & Xiong, L. (2018) Integrative regulation of drought escape through ABA‐dependent and ‐independent pathways in Rice. Molecular Plant, 11, 584–597. 10.1016/j.molp.2018.01.004 29366830

[tpj71180-bib-0016] Fang, X. , Wu, Z. , Raitskin, O. , Webb, K. , Voigt, P. , Lu, T. et al. (2020) The 3′ processing of antisense RNAs physically links to chromatin‐based transcriptional control. PNAS, 117, 15316–15321. 10.1073/pnas.2007268117 PMC7334503 32541063

[tpj71180-bib-0017] Gaude, A.A. , Siqueira, R.H. , Botelho, S.B. & Jalmi, S.K. (2024) Epigenetic arsenal for stress mitigation in plants. Biochimica et Biophysica Acta, General Subjects, 1868, 130620. 10.1016/j.bbagen.2024.130620 38636616

[tpj71180-bib-0018] Guo, L. , Yu, Y. , Law, J.A. & Zhang, X. (2010) SET DOMAIN GROUP2 is the major histone H3 lysine [corrected] 4 trimethyltransferase in Arabidopsis. Proceedings of the National Academy of Sciences of the United States of America, 107, 18557–18562. 10.1073/pnas.1010478107 PMC2972934 20937886

[tpj71180-bib-0019] Hayot, C.M. , Forouzesh, E. , Goel, A. , Avramova, Z. & Turner, J.A. (2012) Viscoelastic properties of cell walls of single living plant cells determined by dynamic nanoindentation. Journal of Experimental Botany, 63, 2525–2540. 10.1093/jxb/err428 PMC3346220 22291130

[tpj71180-bib-0020] Hu, H. & Du, J. (2022) Structure and mechanism of histone methylation dynamics in Arabidopsis. Current Opinion in Plant Biology, 67, 102211. 10.1016/j.pbi.2022.102211 35452951

[tpj71180-bib-0021] Hu, Y. , Jiang, Y. , Han, X. , Wang, H. , Pan, J. & Yu, D. (2017) Jasmonate regulates leaf senescence and tolerance to cold stress: crosstalk with other phytohormones. Journal of Experimental Botany, 68, 1361–1369. 10.1093/jxb/erx004 28201612

[tpj71180-bib-0022] Hwang, K. , Susila, H. , Nasim, Z. , Jung, J.‐Y. & Ahn, J.H. (2019) Arabidopsis ABF3 and ABF4 transcription factors act with the NF‐YC complex to regulate SOC1 expression and mediate drought‐accelerated flowering. Molecular Plant, 12, 489–505. 10.1016/j.molp.2019.01.002 30639313

[tpj71180-bib-0023] Jiang, D. , Wang, Y. , Wang, Y. & He, Y. (2008) Repression of FLOWERING LOCUS C and FLOWERING LOCUS T by the Arabidopsis Polycomb repressive complex 2 components. PLoS One, 3, e3404. 10.1371/journal.pone.0003404 PMC2561057 18852898

[tpj71180-bib-0024] Jiang, Y. , Jiang, S. & Liu, L. (2025) Understanding the multifaceted role of ABA signaling in orchestrating plant developmental transition. Stress Biology, 5, 5.

[tpj71180-bib-0025] Kim, S.Y. , He, Y. , Jacob, Y. , Noh, Y.‐S. , Michaels, S. & Amasino, R. (2005) Establishment of the vernalization‐responsive, Winter‐annual habit in Arabidopsis requires a putative histone H3 methyl transferase. The Plant Cell, 17, 3301–3310. 10.1105/tpc.105.034645 PMC1315370 16258034

[tpj71180-bib-0026] Ko, J.‐H. , Prassinos, C. , Keathley, D. & Han, K.‐H. (2011) Novel aspects of transcriptional regulation in the winter survival and maintenance mechanism of poplar. Tree Physiology, 31, 208–225. 10.1093/treephys/tpq109 21383024

[tpj71180-bib-0027] Lee, C.‐M. & Thomashow, M.F. (2012) Photoperiodic regulation of the C‐repeat binding factor (CBF) cold acclimation pathway and freezing tolerance in Arabidopsis thaliana. PNAS, 109, 15054–15059. 10.1073/pnas.1211295109 PMC3443188 22927419

[tpj71180-bib-0028] Lee, H. , Suh, S.‐S. , Park, E. , Cho, E. , Ahn, J.H. , Kim, S.‐G. et al. (2000) The AGAMOUS‐LIKE 20 MADS domain protein integrates floral inductive pathways in Arabidopsis. Genes & Development, 14, 2366–2376. 10.1101/gad.813600 PMC316936 10995392

[tpj71180-bib-0029] Lee, J. , Yun, J.‐Y. , Zhao, W. , Shen, W.‐H. & Amasino, R.M. (2015) A methyltransferase required for proper timing of the vernalization response in Arabidopsis. PNAS, 112, 2269–2274. 10.1073/pnas.1423585112 PMC4343098 25605879

[tpj71180-bib-0030] Liu, B. , Wei, G. , Shi, J. , Jin, J. , Shen, T. , Ni, T. et al. (2016) SET DOMAIN GROUP 708, a histone H3 lysine 36‐specific methyltransferase, controls flowering time in rice (Oryza sativa). New Phytologist, 210, 577–588. 10.1111/nph.13768 26639303

[tpj71180-bib-0031] Liu, B. , Berr, A. , Chang, C. , Liu, C. , Shen, W.‐H. & Ruan, Y. (2016) Interplay of the histone methyltransferases SDG8 and SDG26 in the regulation of transcription and plant flowering and development. Biochimica et Biophysica Acta (BBA) ‐ Gene Regulatory Mechanisms, 1859, 581–590. 10.1016/j.bbagrm.2016.02.003 26854085

[tpj71180-bib-0032] Naika, M. , Shameer, K. , Mathew, O.K. , Gowda, R. & Sowdhamini, R. (2013) STIFDB2: an updated version of plant stress‐responsive transcription factor database with additional stress signals, stress‐responsive transcription factor binding sites and stress‐responsive genes in Arabidopsis and rice. Plant and Cell Physiology, 54, e8. 10.1093/pcp/pcs185 PMC3583027 23314754

[tpj71180-bib-0033] Pajoro, A. , Severing, E. , Angenent, G.C. & Immink, R.G.H. (2017) Histone H3 lysine 36 methylation affects temperature‐induced alternative splicing and flowering in plants. Genome Biology, 18, 102. 10.1186/s13059-017-1235-x PMC5452352 28566089

[tpj71180-bib-0034] Pareek, A. , Khurana, A. , Sharma, A.K. & Kumar, R. (2017) An overview of signaling regulons during cold stress tolerance in plants. Current Genomics, 18, 498–511. 10.2174/1389202918666170228141345 PMC5684653 29204079

[tpj71180-bib-0035] Phukan, U.J. , Jeena, G.S. & Shukla, R.K. (2016) WRKY transcription factors: molecular regulation and stress responses in plants. Frontiers in Plant Science, 7. Available at: 760. Available from: https://www.ncbi.nlm.nih.gov/pmc/articles/PMC4891567/ [Accessed September 7, 2020. 10.3389/fpls.2016.00760 PMC4891567 27375634

[tpj71180-bib-0036] Pien, S. , Fleury, D. , Mylne, J.S. , Crevillen, P. , Inzé, D. , Avramova, Z. et al. (2008) ARABIDOPSIS TRITHORAX1 dynamically regulates FLOWERING LOCUS C activation via histone 3 lysine 4 trimethylation. The Plant Cell, 20, 580–588. 10.1105/tpc.108.058172 PMC2329943 18375656

[tpj71180-bib-0037] Puig, S. , Mira, H. , Dorcey, E. , Sancenón, V. , Andrés‐Colás, N. , Garcia‐Molina, A. et al. (2007) Higher plants possess two different types of ATX1‐like copper chaperones. Biochemical and Biophysical Research Communications, 354, 385–390. 10.1016/j.bbrc.2006.12.215 17223078

[tpj71180-bib-0038] Qi, P.‐L. , Zhou, H.‐R. , Zhao, Q.‐Q. , Feng, C. , Ning, Y.Q. , Su, Y.N. et al. (2022) Characterization of an autonomous pathway complex that promotes flowering in Arabidopsis. Nucleic Acids Research, 50, 7380–7395. 10.1093/nar/gkac551 PMC9303297 35766439

[tpj71180-bib-0039] Ramirez‐Prado, J.S. , Piquerez, S.J.M. , Bendahmane, A. , Hirt, H. , Raynaud, C. & Benhamed, M. (2018) Modify the histone to win the Battle: chromatin dynamics in plant–pathogen interactions. Frontiers in Plant Science, 9, 355. 10.3389/fpls.2018.00355 PMC5868138 29616066

[tpj71180-bib-0040] Regnault, T. , Davière, J.‐M. , Wild, M. , Sakvarelidze‐Achard, L. , Heintz, D. , Carrera Bergua, E. et al. (2015) The gibberellin precursor GA12 acts as a long‐distance growth signal in Arabidopsis. Nature Plants, 1, 15073. 10.1038/nplants.2015.73 27250008

[tpj71180-bib-0041] Riboni, M. , Robustelli Test, A. , Galbiati, M. , Tonelli, C. & Conti, L. (2016) ABA‐dependent control of GIGANTEA signalling enables drought escape via up‐regulation of FLOWERING LOCUS T in Arabidopsis thaliana. Journal of Experimental Botany, 67, 6309–6322. 10.1093/jxb/erw384 PMC5181575 27733440

[tpj71180-bib-0042] Roitinger, E. , Hofer, M. , Köcher, T. , Pichler, P. , Novatchkova, M. , Yang, J. et al. (2015) Quantitative phosphoproteomics of the ataxia telangiectasia‐mutated (ATM) and ataxia telangiectasia‐mutated and rad3‐related (ATR) dependent DNA damage response in Arabidopsis thaliana. Molecular & Cellular Proteomics, 14, 556–571. 10.1074/mcp.M114.040352 PMC4349977 25561503

[tpj71180-bib-0043] Saleh, A. , Al‐Abdallat, A. , Ndamukong, I. , Alvarez‐Venegas, R. & Avramova, Z. (2007) The Arabidopsis homologs of trithorax (ATX1) and enhancer of zeste (CLF) establish ‘bivalent chromatin marks’ at the silent AGAMOUS locus. Nucleic Acids Research, 35, 6290–6296. 10.1093/nar/gkm464 PMC2094062 17881378

[tpj71180-bib-0044] Seo, E. , Lee, H. , Jeon, J. , Park, H. , Kim, J. , Noh, Y.‐S. et al. (2009) Crosstalk between cold response and flowering in Arabidopsis is mediated through the flowering‐time gene SOC1 and its upstream negative regulator FLC. The Plant Cell, 21, 3185–3197. 10.1105/tpc.108.063883 PMC2782271 19825833

[tpj71180-bib-0045] Shi, Y. , Ding, Y. & Yang, S. (2015) Cold signal transduction and its interplay with phytohormones during cold acclimation. Plant & Cell Physiology, 56, 7–15. 10.1093/pcp/pcu115 25189343

[tpj71180-bib-0046] Shi, Y. , Tian, S. , Hou, L. , Huang, X. , Zhang, X. , Guo, H. et al. (2012) Ethylene signaling negatively regulates freezing tolerance by repressing expression of CBF and type‐a ARR genes in Arabidopsis. Plant Cell, 24, 2578–2595. 10.1105/tpc.112.098640 PMC3406918 22706288

[tpj71180-bib-0047] Smirnova, E. , Marquis, V. , Poirier, L. , Aubert, Y. , Zumsteg, J. , Ménard, R. et al. (2017) Jasmonic acid oxidase 2 hydroxylates Jasmonic acid and represses basal defense and resistance responses against *Botrytis cinerea* infection. Molecular Plant, 10, 1159–1173. 10.1016/j.molp.2017.07.010 28760569

[tpj71180-bib-0048] Springer, N.M. , Napoli, C.A. , Selinger, D.A. , Pandey, R. , Cone, K.C. , Chandler, V.L. et al. (2003) Comparative analysis of SET domain proteins in maize and Arabidopsis reveals multiple duplications preceding the divergence of monocots and dicots. Plant Physiology, 132, 907–925. 10.1104/pp.102.013722 PMC167030 12805620

[tpj71180-bib-0049] Sui, P. , Shi, J. , Gao, X. , Shen, W.‐H. & Dong, A. (2013) H3K36 methylation is involved in promoting Rice flowering. Molecular Plant, 6, 975–977. 10.1093/mp/sss152 23239829

[tpj71180-bib-0050] Tamada, Y. , Yun, J.‐Y. , Woo, S.C. & Amasino, R.M. (2009) ARABIDOPSIS TRITHORAX‐RELATED7 is required for methylation of lysine 4 of histone H3 and for transcriptional activation of FLOWERING LOCUS C. Plant Cell, 21, 3257–3269. 10.1105/tpc.109.070060 PMC2782277 19855050

[tpj71180-bib-0051] Valencia‐Morales, M.d.P. , Camas‐Reyes, J.A. , Cabrera‐Ponce, J.L. & Alvarez‐Venegas, R. (2012) The Arabidopsis thaliana SET‐domain‐containing protein ASHH1/SDG26 interacts with itself and with distinct histone lysine methyltransferases. Journal of Plant Research, 125, 679–692. 10.1007/s10265-012-0485-7 22438063

[tpj71180-bib-0052] Vishwakarma, K. , Upadhyay, N. , Kumar, N. et al. (2017) Abscisic acid signaling and abiotic stress tolerance in plants: a review on current knowledge and future prospects. Frontiers in Plant Science, 8, 161. 10.3389/fpls.2017.00161 PMC5316533 28265276

[tpj71180-bib-0053] Winter, D. , Vinegar, B. , Naef, H. , Ammar, R. , Wilson, G.N. & Provart, N.J. (2007) An “electronic fluorescent pictograph” browser for exploring and analyzing large‐scale biological data sets. PLoS One, 2, e718. 10.1371/journal.pone.0000718 PMC1934936 17684564

[tpj71180-bib-0054] Xu, C. , Fang, X. , Lu, T. & Dean, C. (2021) Antagonistic cotranscriptional regulation through ARGONAUTE1 and the THO/TREX complex orchestrates FLC transcriptional output. Proceedings of the National Academy of Sciences of the United States of America, 118, e2113757118. 10.1073/pnas.2113757118 PMC8617408 34789567

[tpj71180-bib-0055] Xu, L. , Zhao, Z. , Dong, A. , Soubigou‐Taconnat, L. , Renou, J.‐P. , Steinmetz, A. et al. (2008) Di‐ and tri‐ but not Monomethylation on histone H3 lysine 36 Marks active transcription of genes involved in flowering time regulation and other processes in Arabidopsis thaliana. Molecular and Cellular Biology, 28, 1348–1360. 10.1128/MCB.01607-07 PMC2258740 18070919

[tpj71180-bib-0056] Yao, Y. , Zhou, J. , Wang, J. , Lei, X. , Jiang, A. & Sun, Q. (2025) H3K36 methylation stamps transcription resistive to preserve development in plants. Nature Plants, 11, 808–820. 10.1038/s41477-025-01962-6 40164787

[tpj71180-bib-0057] Yu, M.‐H. , Liao, W.‐C. & Wu, K. (2025) Histone methylation in plant responses to abiotic stresses. Journal of Experimental Botany, 76, 4771–4886. 10.1093/jxb/eraf058 39945718

[tpj71180-bib-0058] Zeng, J. , Yang, L. , Tian, M. , Xie, X. , Liu, C. & Ruan, Y. (2023) SDG26 is involved in trichome control in Arabidopsis thaliana: affecting phytohormones and adjusting accumulation of H3K27me3 on genes related to trichome growth and development. Plants (Basel, Switzerland), 12, 1651. 10.3390/plants12081651 PMC10143075 37111875

[tpj71180-bib-0059] Zhang, H. , Zhu, J. , Gong, Z. & Zhu, J.‐K. (2022) Abiotic stress responses in plants. Nature Reviews. Genetics, 23, 104–119. 10.1038/s41576-021-00413-0 34561623

[tpj71180-bib-0060] Zhang, J.Z. , Creelman, R.A. & Zhu, J.‐K. (2004) From laboratory to field. Using information from Arabidopsis to engineer salt, cold, and drought tolerance in crops. Plant Physiology, 135, 615–621. 10.1104/pp.104.040295 PMC514097 15173567

[tpj71180-bib-0061] Zhang, X. , Ménard, R. , Li, Y. , Coruzzi, G.M. , Heitz, T. , Shen, W.‐H. et al. (2020) Arabidopsis SDG8 potentiates the sustainable transcriptional induction of the pathogenesis‐related genes PR1 and PR2 during plant defense response. Frontiers in Plant Science, 11, 277. 10.3389/fpls.2020.00277 PMC7078350 32218796

[tpj71180-bib-0062] Zhao, Z. , Yu, Y. , Meyer, D. , Wu, C. & Shen, W.‐H. (2005) Prevention of early flowering by expression of FLOWERING LOCUS C requires methylation of histone H3 K36. Nature Cell Biology, 7, 1256–1260. 10.1038/ncb1329 16299497

