# Supplementary material for: SDG26 integrates ABA‐dependent and ABA‐independent pathways to regulate cold stress responses in Arabidopsis via chromatin modification
Source: Plant J. 2026 Oct 23;128(2):e71180. doi: 10.1111/tpj.71180 (PMC13691520; doi:10.1111/tpj.71180)
Supplement: Supplementary file 2 [file TPJ-128-0-s003.pdf]

S5

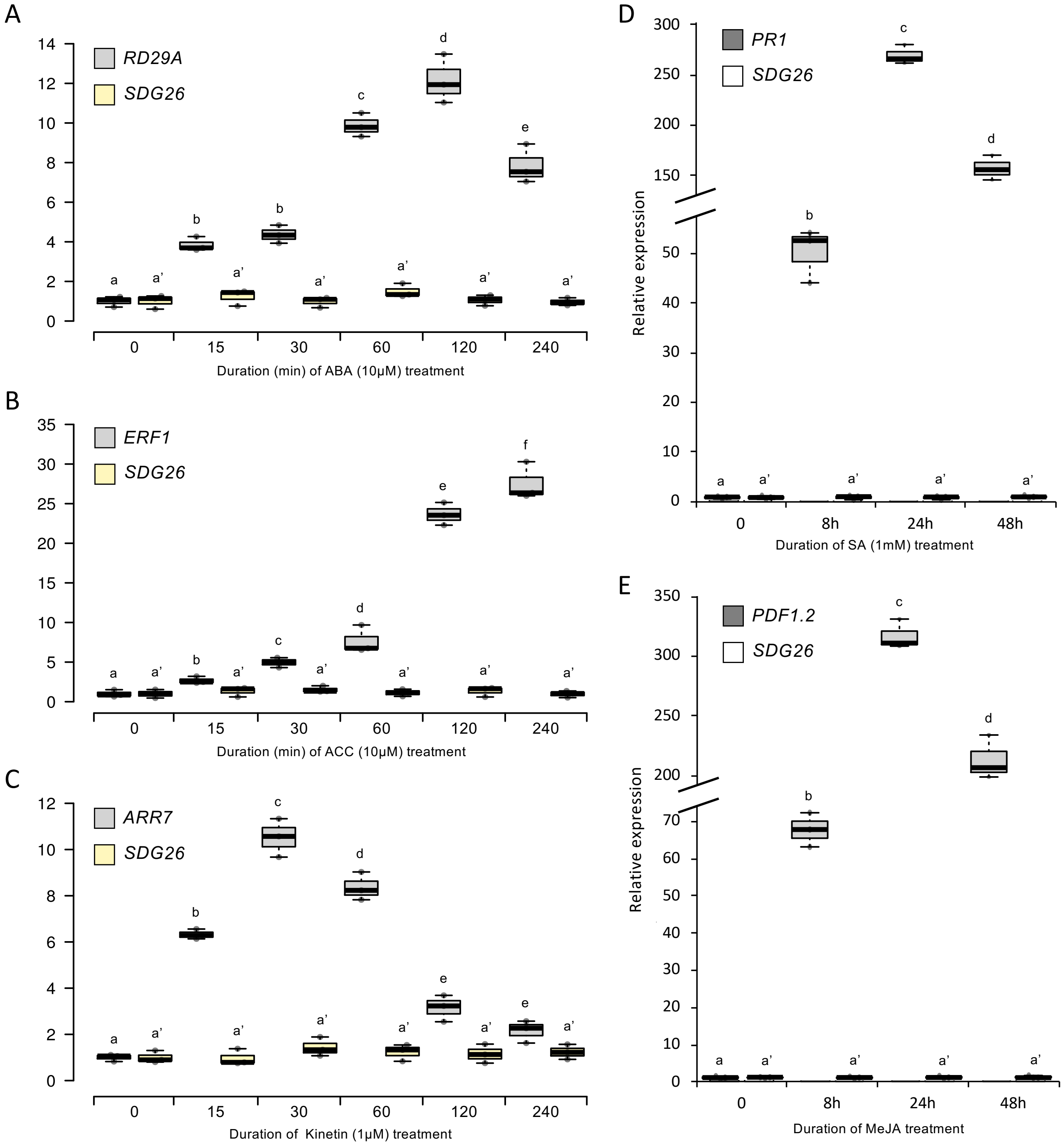
A
RD29A
SDG26
Duration (min) of ABA (10µM) treatment
B
ERF1
SDG26
Duration (min) of ACC (10µM) treatment
C
ARR7
SDG26
Duration of Kinetin (1µM) treatment
D
PR1
SDG26
Relative expression
Duration of SA (1mM) treatment
E
PDF1.2
SDG26
Relative expression
Duration of MeJA treatment
