# Supplementary material for: SDG26 integrates ABA‐dependent and ABA‐independent pathways to regulate cold stress responses in Arabidopsis via chromatin modification
Source: Plant J. 2026 Oct 23;128(2):e71180. doi: 10.1111/tpj.71180 (PMC13691520; doi:10.1111/tpj.71180)
Supplement: Supplementary file 3 [file TPJ-128-0-s004.pdf]

S9

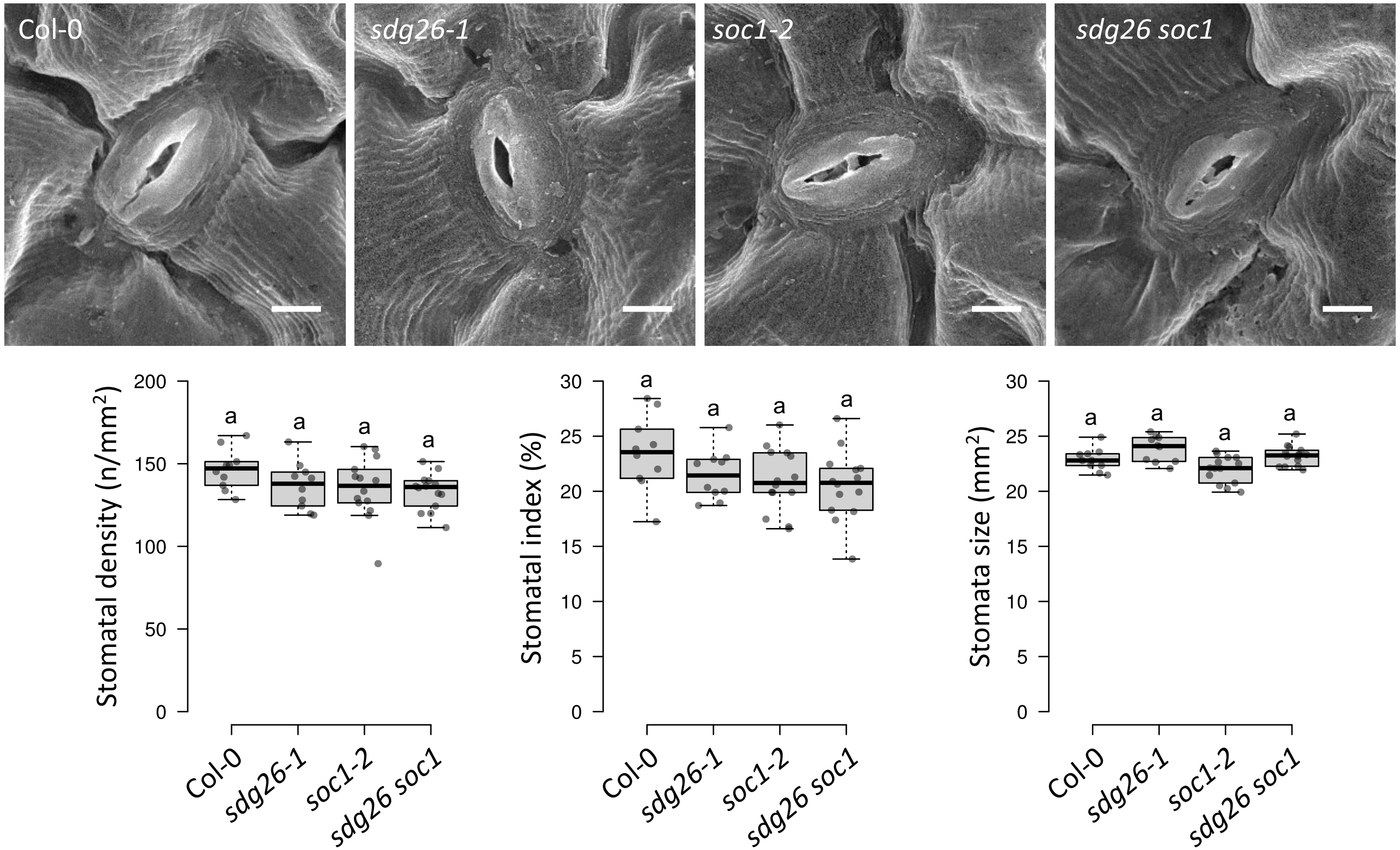
Col-0
sdg26-1
soc1-2
sdg26 soc1
Stomatal density (n/mm2)
200
150
100
50
0
a
a
a
a
Col-0
sdg26-1
soc1-2
sdg26 soc1
Stomatal index (%)
30
25
20
15
10
5
0
a
a
a
a
Col-0
sdg26-1
soc1-2
sdg26 soc1
Stomata size (mm2)
30
25
20
15
10
5
0
a
a
a
a
Col-0
sdg26-1
soc1-2
sdg26 soc1
