# Supplementary material for: SDG26 integrates ABA‐dependent and ABA‐independent pathways to regulate cold stress responses in Arabidopsis via chromatin modification
Source: Plant J. 2026 Oct 23;128(2):e71180. doi: 10.1111/tpj.71180 (PMC13691520; doi:10.1111/tpj.71180)
Supplement: Supplementary file 4 [file TPJ-128-0-s005.pdf]

S8

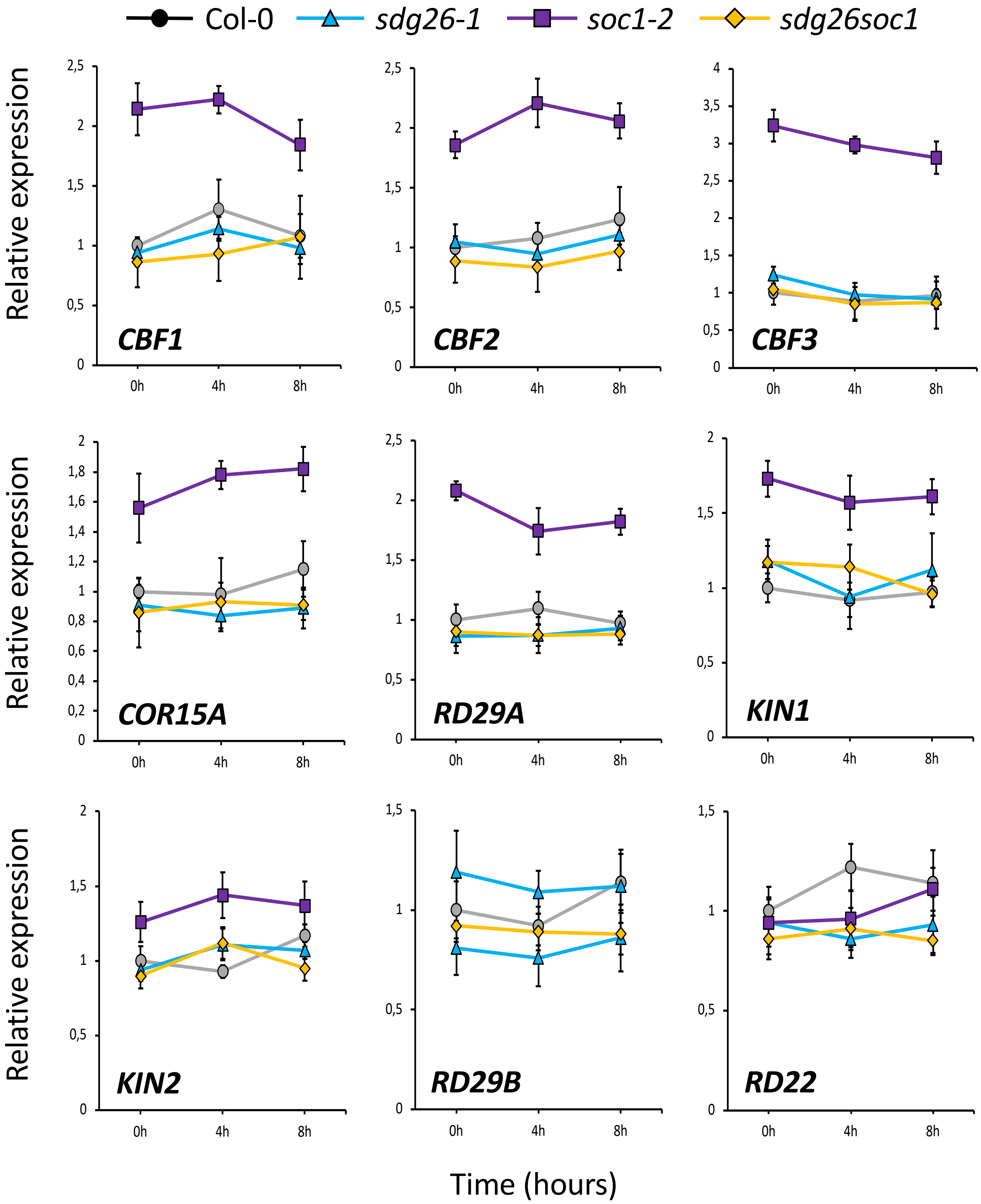

Col-0
sdg26-1
soc1-2
sdg26soc1
Relative expression
CBF1
CBF2
CBF3
COR15A
RD29A
KIN1
KIN2
RD29B
RD22
0h
4h
8h
Time (hours)
