# Supplementary material for: SDG26 integrates ABA‐dependent and ABA‐independent pathways to regulate cold stress responses in Arabidopsis via chromatin modification
Source: Plant J. 2026 Oct 23;128(2):e71180. doi: 10.1111/tpj.71180 (PMC13691520; doi:10.1111/tpj.71180)
Supplement: Supplementary file 5 [file TPJ-128-0-s006.pdf]

S4

A

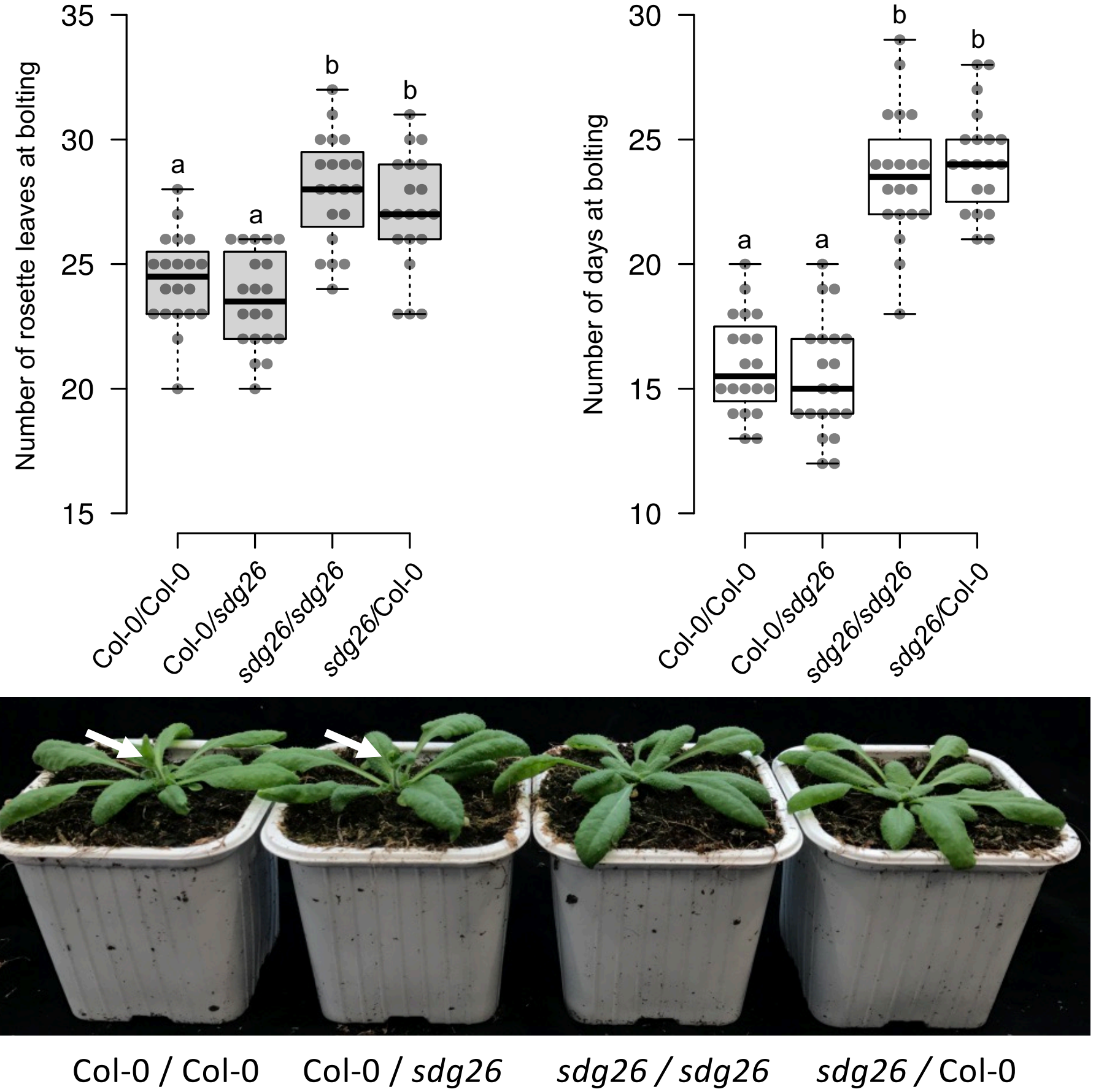

Number of rosette leaves at bolting
35
30
25
20
15
a
a
b
b
Col-0/Col-0
Col-0/sdg26
sdg26/sdg26
sdg26/Col-0
Number of days at bolting
30
25
20
15
10
a
a
b
b
Col-0/Col-0
Col-0/sdg26
sdg26/sdg26
sdg26/Col-0
Col-0 / Col-0
Col-0 / sdg26
sdg26 / sdg26
sdg26 / Col-0

B

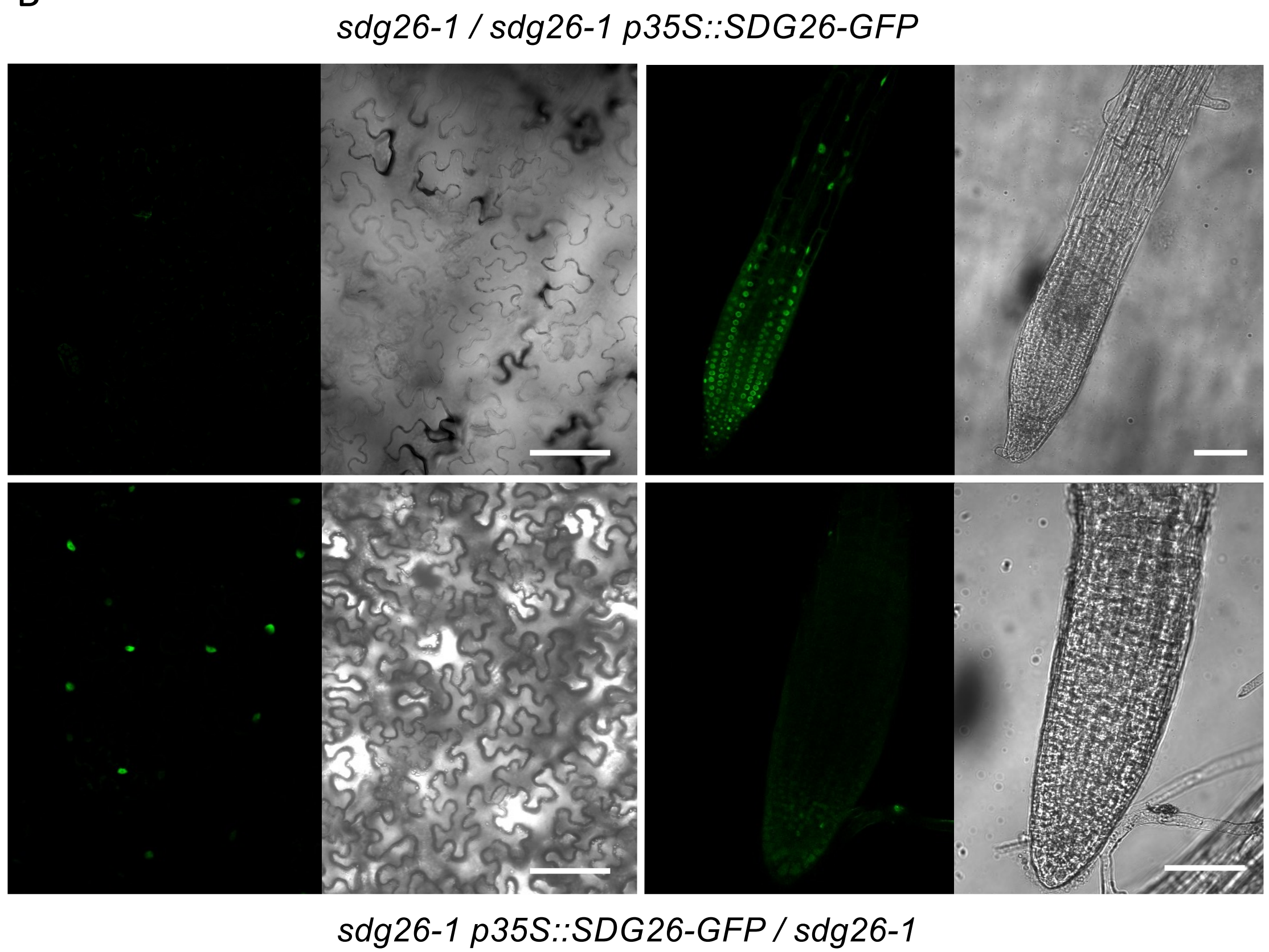

sdg26-1 / sdg26-1 p35S::SDG26-GFP
sdg26-1 p35S::SDG26-GFP / sdg26-1
