# Supplementary material for: SDG26 integrates ABA‐dependent and ABA‐independent pathways to regulate cold stress responses in Arabidopsis via chromatin modification
Source: Plant J. 2026 Oct 23;128(2):e71180. doi: 10.1111/tpj.71180 (PMC13691520; doi:10.1111/tpj.71180)
Supplement: Supplementary file 6 [file TPJ-128-0-s007.pdf]

S7

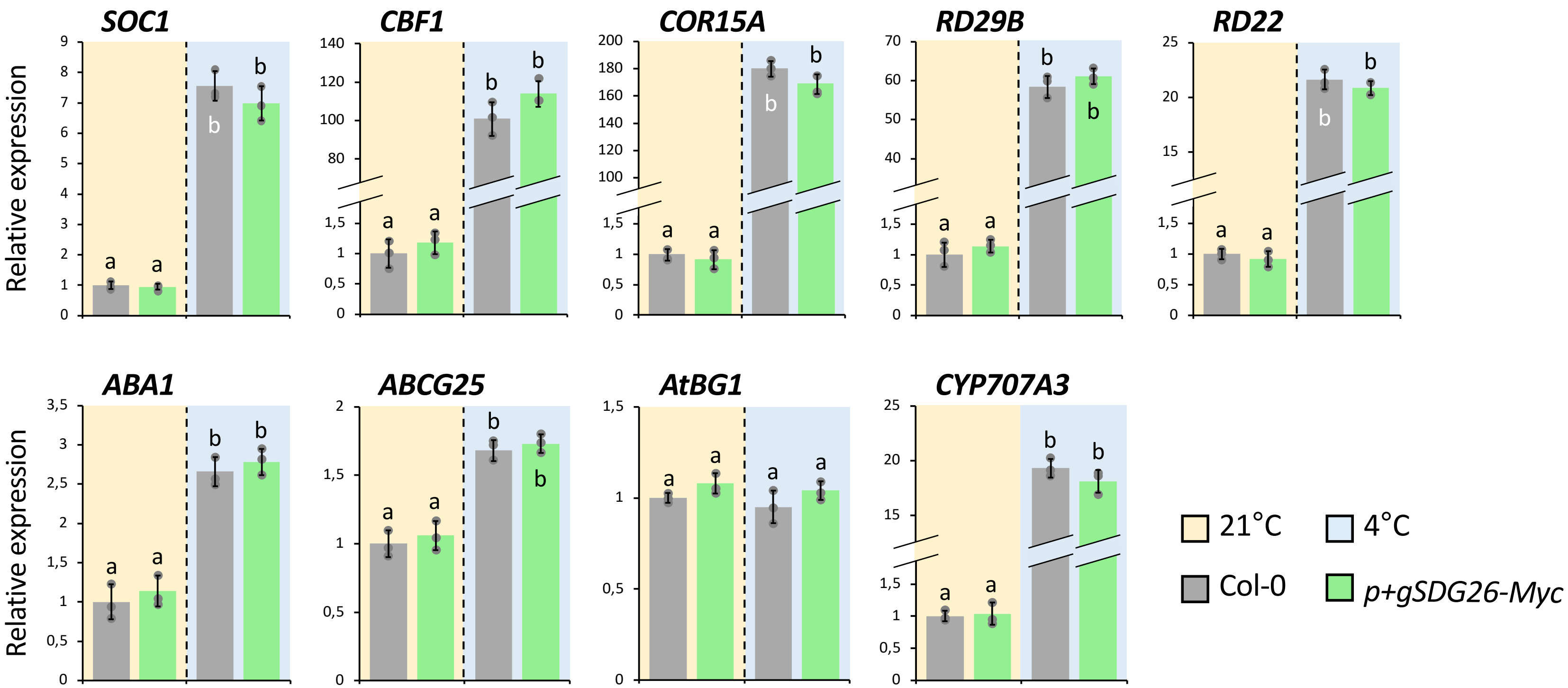

SOC1
CBF1
COR15A
RD29B
RD22
ABA1
ABCG25
AtBG1
CYP707A3
Relative expression
Relative expression
21°C
4°C
Col-0
p+gSDG26-Myc
