# Supplementary material for: SDG26 integrates ABA‐dependent and ABA‐independent pathways to regulate cold stress responses in Arabidopsis via chromatin modification
Source: Plant J. 2026 Oct 23;128(2):e71180. doi: 10.1111/tpj.71180 (PMC13691520; doi:10.1111/tpj.71180)
Supplement: Supplementary file 8 [file TPJ-128-0-s009.pdf]

S10

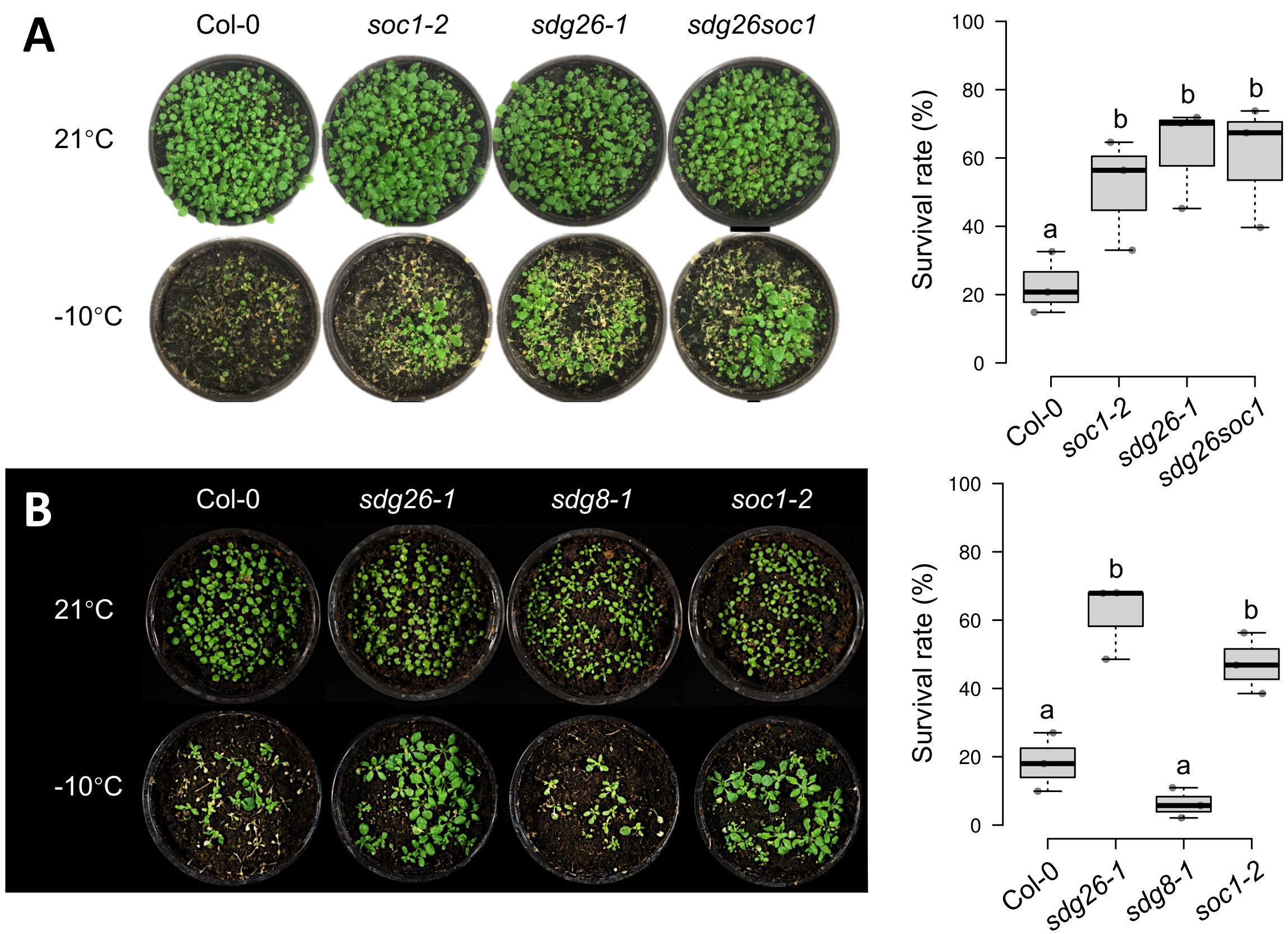
A
Col-0
soc1-2
sdg26-1
sdg26soc1
21°C
-10°C
Survival rate (%)
100
80
60
40
20
0
a
b
b
b
Col-0
soc1-2
sdg26-1
sdg26soc1
B
Col-0
sdg26-1
sdg8-1
soc1-2
21°C
-10°C
Survival rate (%)
100
80
60
40
20
0
a
b
a
b
Col-0
sdg26-1
sdg8-1
soc1-2
