# Supplementary material for: SDG26 integrates ABA‐dependent and ABA‐independent pathways to regulate cold stress responses in Arabidopsis via chromatin modification
Source: Plant J. 2026 Oct 23;128(2):e71180. doi: 10.1111/tpj.71180 (PMC13691520; doi:10.1111/tpj.71180)

S1

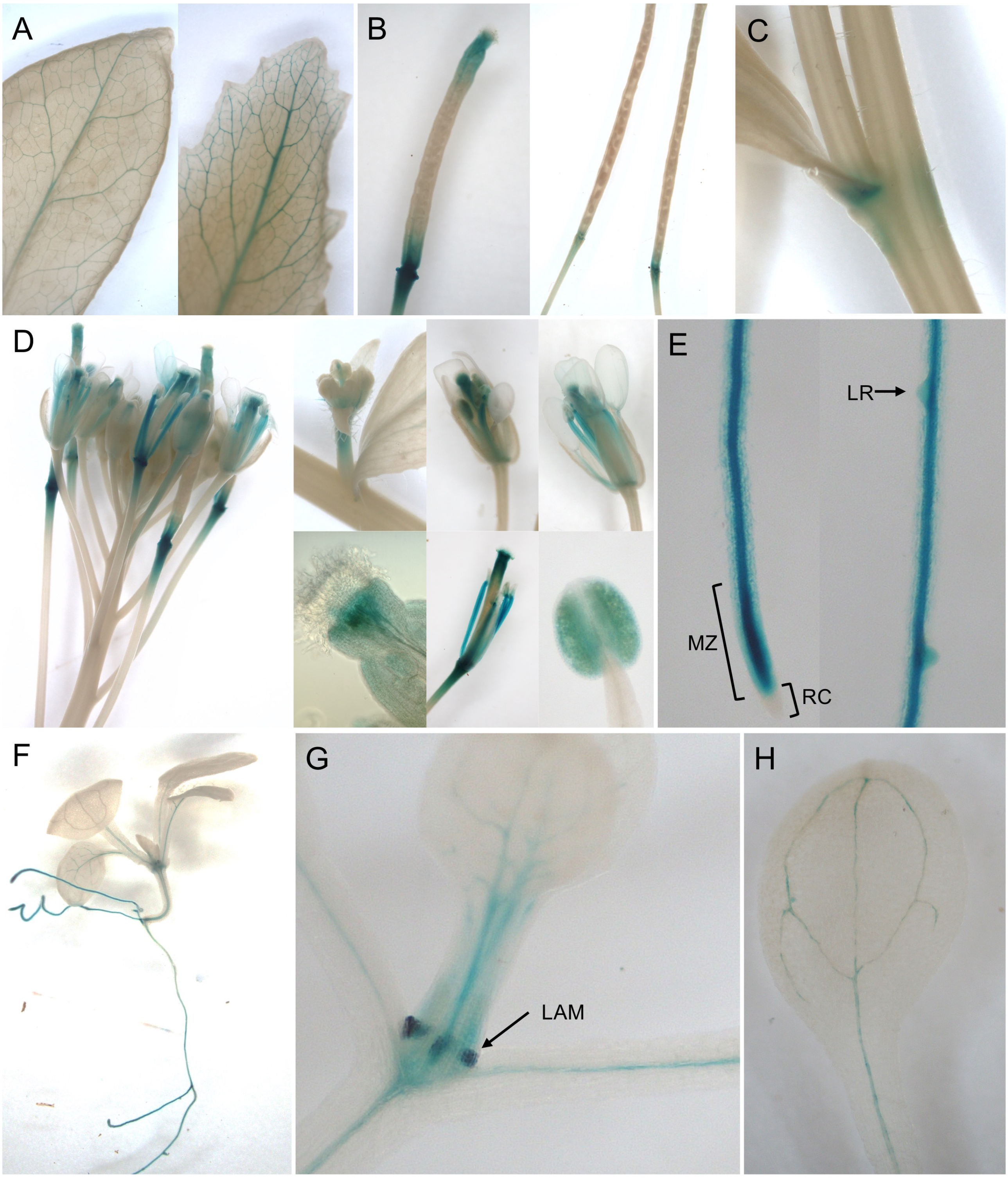

Supplement: Supplementary file 9 [file TPJ-128-0-s011.pdf]
