# Supplementary material for: SDG26 integrates ABA‐dependent and ABA‐independent pathways to regulate cold stress responses in Arabidopsis via chromatin modification
Source: Plant J. 2026 Oct 23;128(2):e71180. doi: 10.1111/tpj.71180 (PMC13691520; doi:10.1111/tpj.71180)
Supplement: Supplementary file 10 [file TPJ-128-0-s012.pdf]

S3

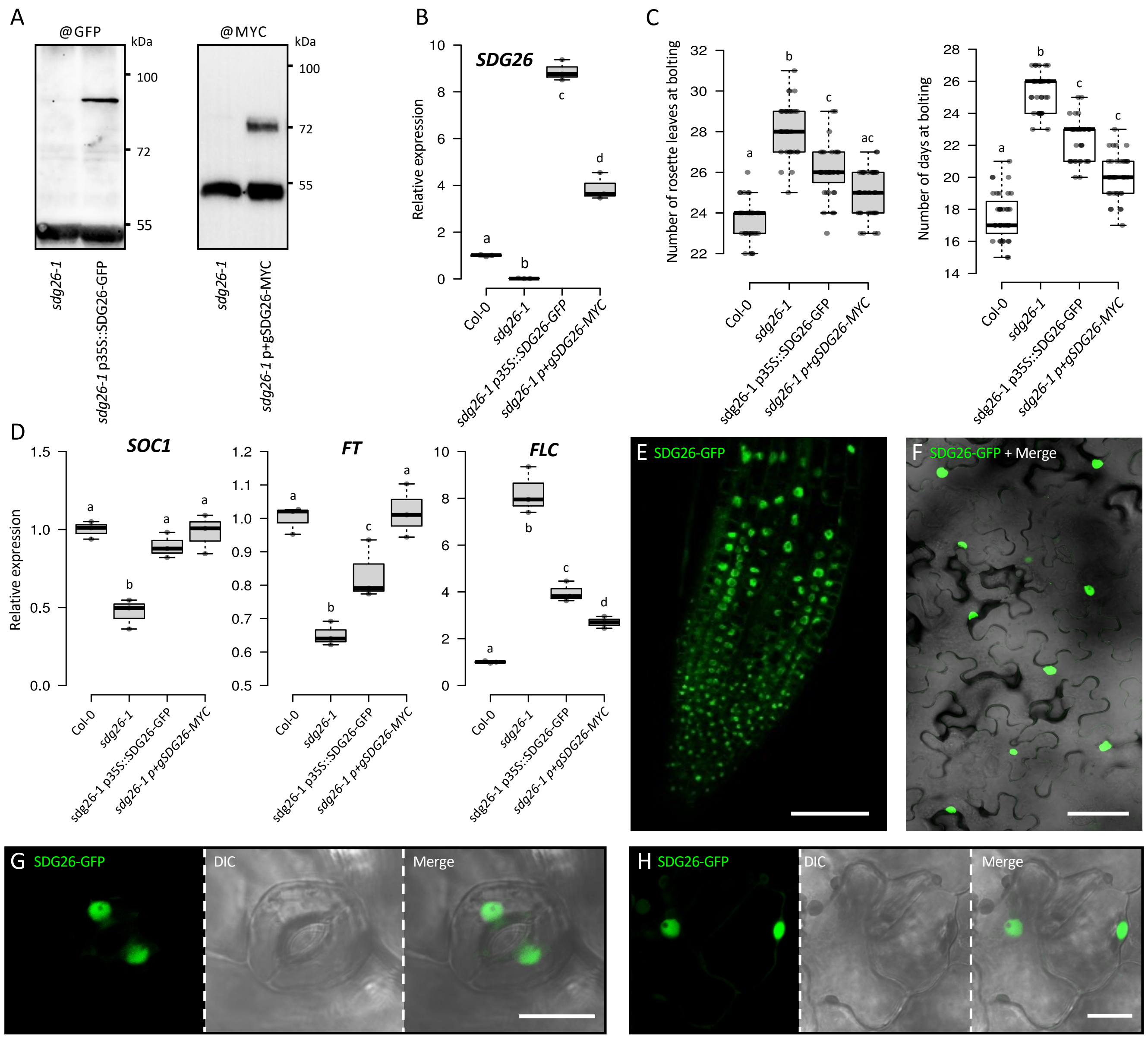

A
@GFP
kDa
100
72
55
sdg26-1
sdg26-1 p35S::SDG26-GFP
@MYC
kDa
100
72
55
sdg26-1
sdg26-1 p+gSDG26-MYC
B
SDG26
Relative expression
Col-0
sdg26-1
sdg26-1 p35S::SDG26-GFP
sdg26-1 p+gSDG26-MYC
C
Number of rosette leaves at bolting
Number of days at bolting
D
SOC1
FT
FLC
Relative expression
E SDG26-GFP
F SDG26-GFP + Merge
G SDG26-GFP
DIC
Merge
H SDG26-GFP
DIC
Merge
